# Clustered Desynchronization from High-Frequency Deep Brain Stimulation

**DOI:** 10.1371/journal.pcbi.1004673

**Published:** 2015-12-29

**Authors:** Dan Wilson, Jeff Moehlis

**Affiliations:** Department of Mechanical Engineering, University of California, Santa Barbara, Santa Barbara, Calfornia, United States of America; University College London, UNITED KINGDOM

## Abstract

While high-frequency deep brain stimulation is a well established treatment for Parkinson’s disease, its underlying mechanisms remain elusive. Here, we show that two competing hypotheses, desynchronization and entrainment in a population of model neurons, may not be mutually exclusive. We find that in a noisy group of phase oscillators, high frequency perturbations can separate the population into multiple clusters, each with a nearly identical proportion of the overall population. This phenomenon can be understood by studying maps of the underlying deterministic system and is guaranteed to be observed for small noise strengths. When we apply this framework to populations of Type I and Type II neurons, we observe clustered desynchronization at many pulsing frequencies.

## Author Summary

While high-frequency deep brain stimulation (DBS) is a decades old treatment for alleviating the motor symptoms Parkinsons disease, the way in which it alleviates these symptoms is not well understood. Making matters more complicated, some experimental results suggest that DBS works by making neurons fire more regularly, while other seemingly contradictory results suggest that DBS works by making neural firing patterns less synchronized. Here we present theoretical and numerical results with the potential to merge these competing hypotheses. For predictable DBS pulsing rates, in the presence of a small amount of noise, a population of neurons will split into distinct clusters, each containing a nearly identical proportion of the overall population. When we observe this clustering phenomenon, on a short time scale, neurons are entrained to high-frequency DBS pulsing, but on a long time scale, they desynchronize predictably.

## Introduction

High frequency deep brain stimulation (DBS), a medical treatment in which high-frequency, pulsatile current is injected into an appropriate brain region, is a well established technique for alleviating tremors, rigidity, and bradykinesia in patients with Parkinson’s disease [1, 2]. While the underlying mechanisms of deep brain stimulation remain unknown, it is well documented that local field potential recordings recorded in the subthalamic nucleus of patients with Parkinson’s disease display increased power in the beta range (approximately 13–35 Hz) [3–5]. These findings have led to the hypothesis that pathological synchronization among neurons in the basal ganglia-cortical loop contribute to the motor symptoms of Parkinson’s disease [6–8]. This hypothesis has been supported by findings that when DBS is applied to the STN, abatement of motor symptoms is correlated with a decrease the power in the beta band of the local field potential recorded from STN [9–11]. This line of thinking has led researchers to develop new strategies for desynchronizing populations of pathologically synchronized oscillators, [12–14], some of which have shown promise as new treatment options for Parkinson’s disease in animal and human studies [15, 16].

While many factors including the location of the DBS probe, stimulus duration, and stimulus magnitude influence the efficacy of DBS, one factor that is difficult to explain is the strong dependency on stimulus frequency. Low-frequency stimulation (≤ 50 Hz) is generally ineffective at reducing symptoms of Parkinson’s disease while high-frequency stimulation from 70 to 1000 Hz and beyond has been shown to be therapeutically effective [17–19]. However, not all high frequency stimulation is equally effective, and clinicians have generally settled on a therapeutic range at about 130–180 Hz. [20, 21].

In an effort to provide insight into the frequency dependent effects of DBS, the authors of [22] postulated that specifically tuned pulse parameters might yield chaotic desynchronization in a network of neurons. If desynchronization is the goal of DBS, then achieving it chaotically is a worthwhile objective. However, this can generally only be seen in a small window of pulse parameters and frequencies which may make it difficult to observe in real neurons. Furthermore, in both brain slices and *in vivo* recordings, individual neuronal spikes have been found to be time-locked to the external high-frequency stimulation [23–28] which would be unlikely if the spike times were chaotic.

Here we present a different viewpoint showing that with high frequency pulsatile stimulation, in the presence of a small amount of noise, a population of neurons can split into distinct clusters, each containing a nearly identical proportion of the overall population. We find that the number of clusters, and hence desynchronization, is highly dependent the pulsing frequency and strength. We provide theoretical insight into this phenomenon and show that it can be observed over a wide range of pulsing frequencies and pulsing strengths. This viewpoint merges two seemingly contradictory hypotheses, showing that the therapeutic effect of the periodic pulsing could be to replace the pathological behavior with a less synchronous pattern of activity, even if individual neuronal spikes are phase locked to the DBS pulses.

## Results

### Clustered Desynchronization in a Computational Neural Network

Consider a noisy, periodically oscillating population of thalamic neurons from [29]:

$$\begin{array}{cc} C\dot{V_{i}} & =f_{V}\left(V_{i},h_{i},r_{i}\right)+I_{b}+u\left(t\right)+\epsilon \eta_{i}\left(t\right),\\ \dot{h_{i}} & =f_{h}\left(V_{i},h_{i}\right),\\ \dot{r_{i}} & =f_{r}\left(V_{i},r_{i}\right),\;i=1,\ldots ,N. \end{array}$$ (1)

Here *V*_*i*_, *h*_*i*_, and *r*_*i*_ represent the transmembrane voltage and gating variables of neuron *i*, respectively, with all functions and parameters taken to be identical to those found in [29], DBS pulses are represented by an external current *u*(*t*), taken to be identical for each neuron, *η*_*i*_(*t*) is a Gaussian white noise process, *C* = 1*μ*F/cm^2^ is the constant neural membrane capacitance, *I_b_* = 1.93*μ*A/*μ*F is a baseline current chosen so that in the absence of external perturbations and noise the firing rate is 60 Hz, and *N* is the total number of neurons.

**Fig 1:**
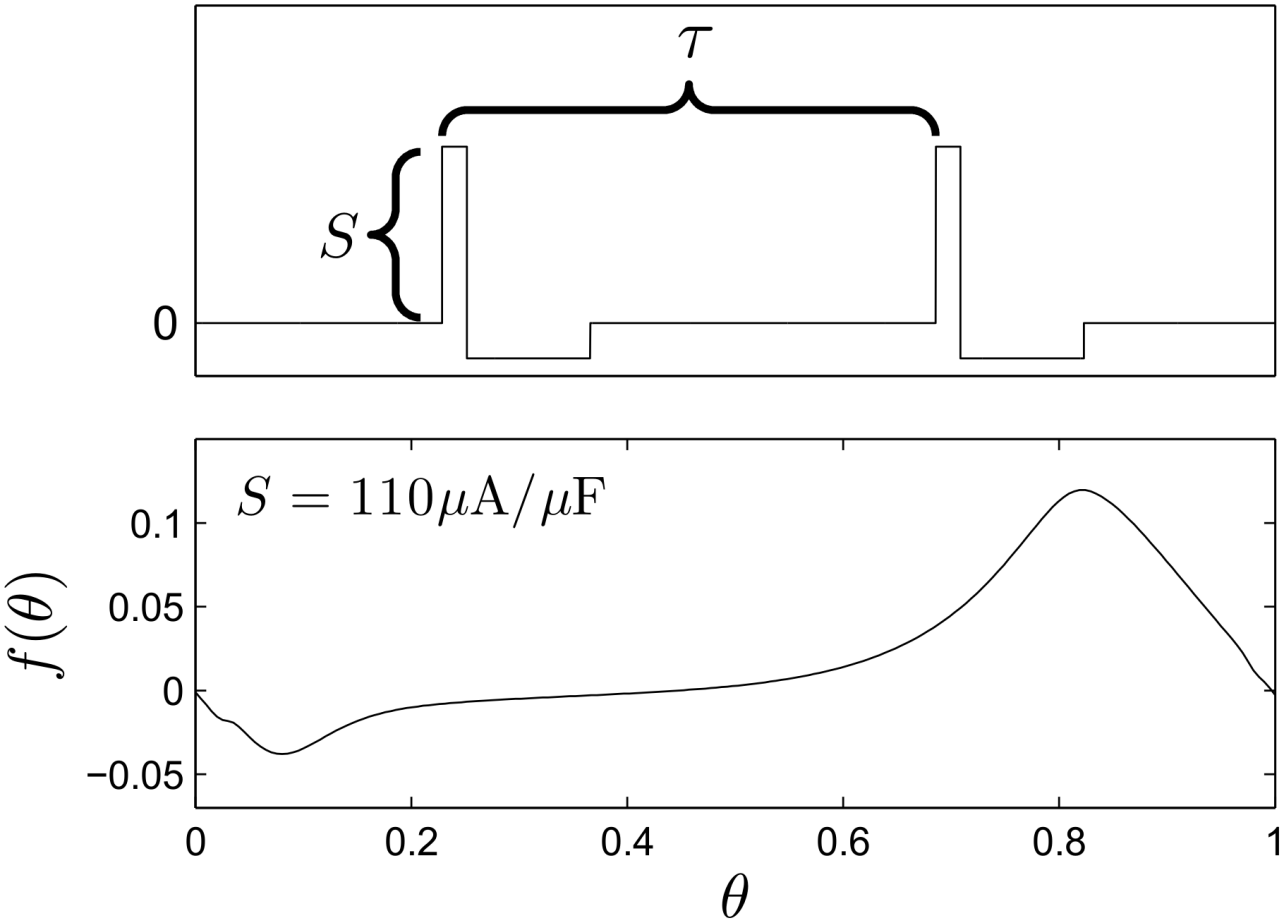
Charge-balanced DBS pulses. The top panel shows a simple, charge-balanced biphasic stimulus chosen to represent a DBS pulse with frequency 1/*τ* and strength *S*. The bottom panel shows the relationship between the phase at the onset of a pulse and the induced change in phase at a particular strength. The effect of the stimulus is generally phase advancing.

Using phase reduction, [30, 31], we can study Eq (1) in a more convenient form:

$$\begin{array}{c} \dot{\theta_{i}}=\omega +f\left(\theta_{i}\right)\delta \left(\text{mod}\left(t,\tau \right)\right)+\epsilon \eta_{i}\left(t\right)Z\left(\theta_{i}\right)+O\left(\epsilon^{2}\right),\;i=1,\ldots ,N, \end{array}$$ (2)

where *θ* ∈ [0, 1) is the phase of the neuron with *θ* = 0 defined to be the time the neuron fires an action potential, *ω* is the natural frequency of oscillation, *f*(*θ*) is a continuous function which describes the effect of the DBS pulse, *τ* is a positive constant that determines the period of the DBS input, and *Z*(*θ*) is the neuron’s phase response curve to an infinitesimal perturbation. Here we assume that *ϵ* is small enough so that higher order noise terms are negligible (c.f. [32, 33]). Fig 1 shows an example charge-balanced pulsatile stimulus. We take the positive portion to be five times larger than the negative portion, with the positive current applied for 100 *μs*. The bottom panel shows the function *f*(*θ*) for a given stimulus intensity, calculated using the direct method [34]: a pulsatile perturbation is applied to a neuron at a known phase *θ*_*p*_ so that *f*(*θ*_*p*_) can be inferred by measuring the timing of the next spike. We note that even though the DBS pulse itself is not a *δ*-function, it is of short enough duration that Eq (2) is an accurate approximation to Eq (1).

**Fig 2:**
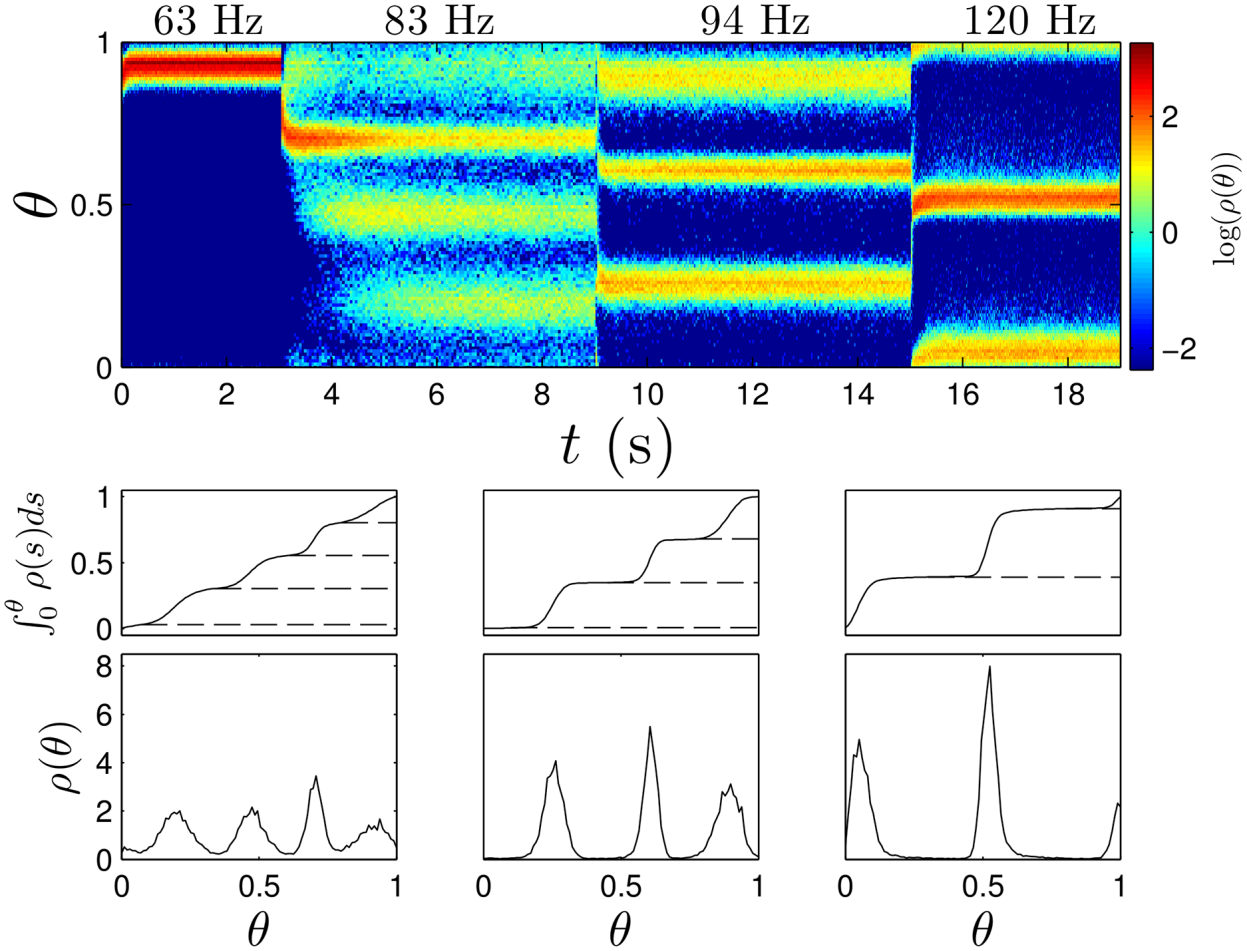
Clustered desynchronization in thalamic neurons. In simulations of Eq (1), the top panel shows snapshots of the probability distribution *ρ*(*θ*) taken immediately preceding every pulse for the 63 Hz stimulation and every fourth, third, and second pulse for the 83 Hz, 94 Hz, and 120 Hz stimulation, respectively. From left to right, the bottom panels show average probability distributions from the final fifty snapshots while stimulating at 83, 94, and 120 Hz respectively. Horizontal dashed lines denote troughs of the probability distributions. The probability contained between successive troughs is 0.27, 0.25, 0.25, and 0.23 in the left panels, 0.34, 0.33, and 0.33 in the middle panels, and 0.52 and 0.48 in the right panels.

**Fig 3:**
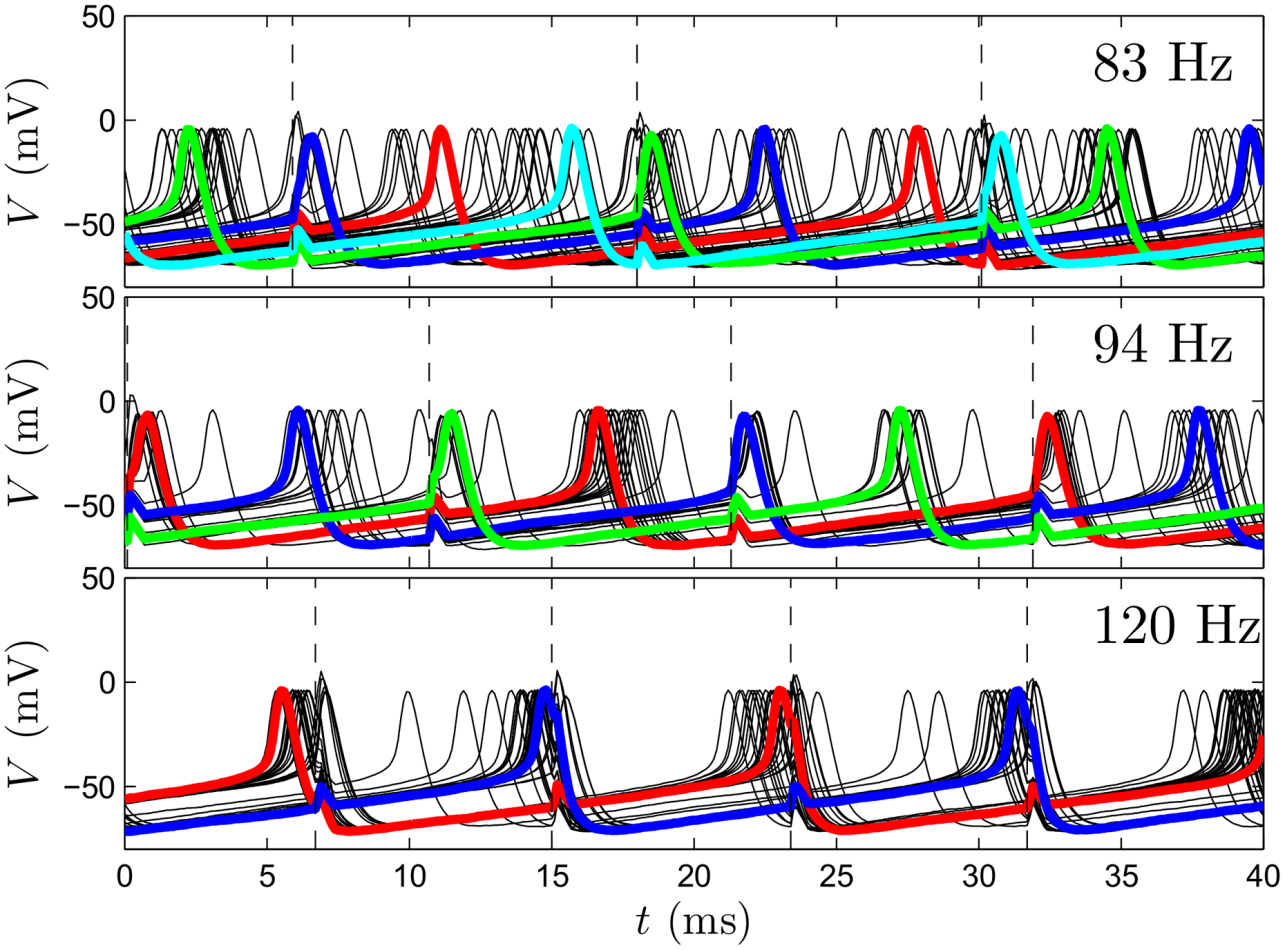
Voltage traces from thalamic neuron population simulations. Top, middle, and bottom panels show clustered states achieved with pulsing at 83, 94, and 120 Hz, respectively. Highlighted traces represent individual neurons from separate clusters. Dashed lines represent the time at which the pulses are applied.

We simulate Eq (1) with 1000 neurons, taking a pulse strength *S* = 110*μ*A/*μ*F, and noise strength $\epsilon =\sqrt{0.05}$, for various pulsing frequencies, with results shown in Fig 2. After some initial transients, we find the network tends to settle to a state with different numbers of clusters for different pulsing frequencies. From the probability distributions of neural phases *ρ*(*θ*), the bottom panels show somewhat surprisingly that once the network settles to a clustered state, each cluster contains a nearly identical portion of the overall population. Also, upon reaching the steady distribution, neurons can still transition between clusters, but on average, the amount that leave a given cluster must be identical to the amount that enter. Fig 3 shows individual voltage traces for 50 sample neurons from this population after the network settles to a clustered state. Highlighted traces represent neurons from each cluster. In general, increasing the number of clusters will decrease synchrony in the population. Furthermore, neurons are more likely to transition between clusters as the overall number of clusters becomes larger.

### A Theoretical Basis for Clustered Desynchronization

For simplicity of notation, we will take *ω* = 1 for Eq (2) in the theoretical analysis, but note that any other value could be considered to obtain qualitatively similar results. In the absence of noise, one may integrate Eq (2) for a single neuron *θ* to yield

$$\begin{array}{c} \theta (t)=\theta (0)+t,\;\;\;\;\;\;\text{for}\;t<\tau ,\\ \theta (t)=\theta (0)+f(\theta (0)+\tau )+t,\;\;\text{for}\;\tau \leq t<2\tau . \end{array}$$ (3)

In this work, we are interested in the state of the system immediately after each pulsatile input. By integrating Eq (2), the system dynamics can be understood in terms of compositions of a map

$$\begin{array}{c} \theta \left(n\tau \right)=g^{\left(n\right)}\left(\theta_{0}\right),\;n=1,2,\ldots , \end{array}$$ (4)

where *g*(*s*) = *s* + *f*(*s* + *τ*) + *τ* and *g* ^(*n*)^ denotes the composition of *g* with itself *n* times, and *θ*_0_ is the initial state of a neuron. In Eqs (3) and (4), *θ*(*t*) and the arguments of *f* and *g* are always evaluated modulo 1. If *g* ^(*n*)^ has a stable fixed point, then any oscillator which starts within its basin of attraction will approach that fixed point as time approaches infinity [35].

**Fig 4:**
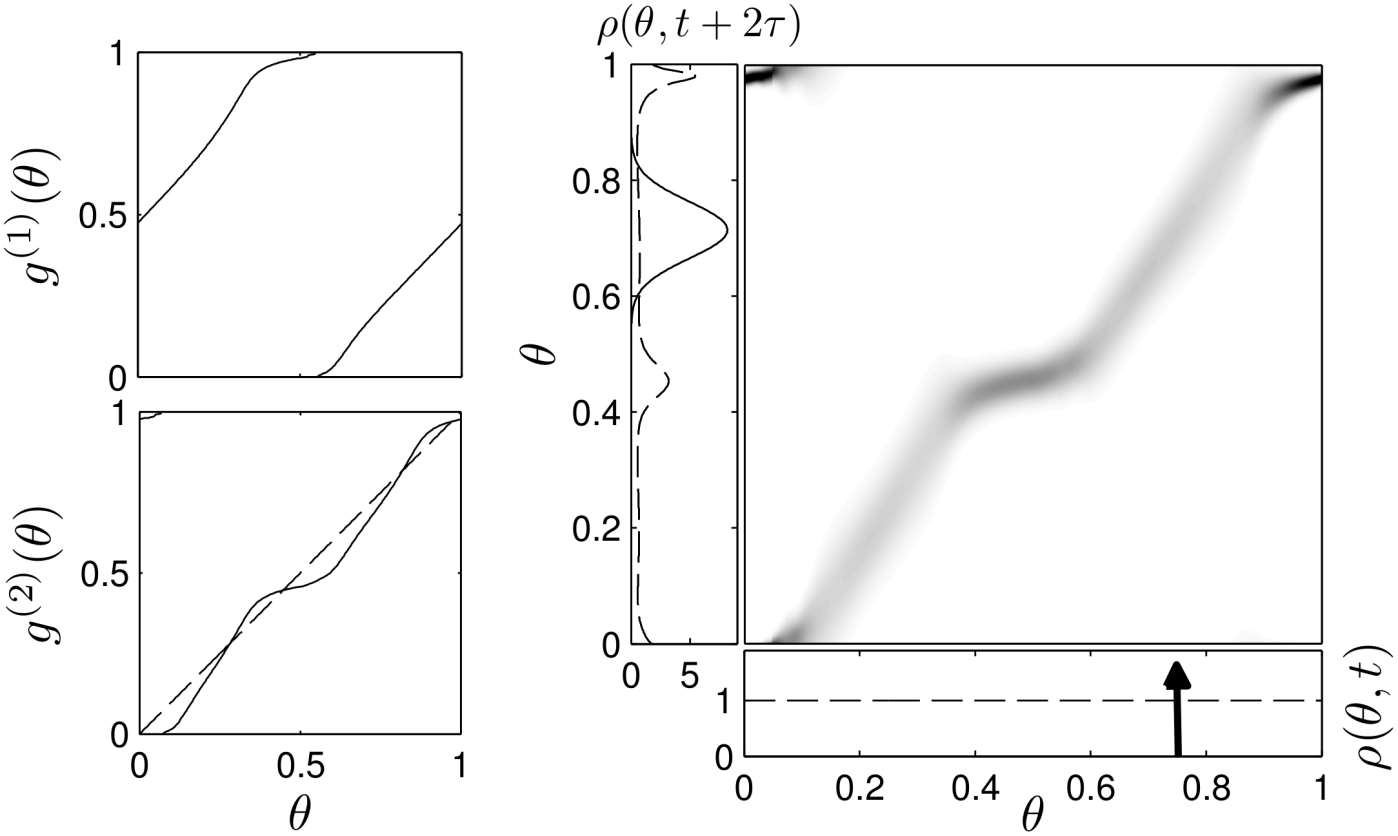
Deterministic and stochastic maps. The left panels show an example map *g* ^(1)^ and *g* ^(2)^, which is obtained from *g* ^(1)^ for a given value of *τ*. The right panel shows stochastic matrix corresponding to *g* ^(2)^, with darker regions representing larger numbers. After 2*τ* has elapsed, the delta function distribution (arrow) is mapped to a Gaussian distribution (solid line) while the uniform distribution is mapped to a bimodal distribution (dashed line).

With noise, the dynamics are more complicated. In this case, the phase of each neuron cannot be determined exactly from Eq (2), but rather, follows a probability distribution. For a neuron with known initial phase *θ*_0_, after *mτ* has elapsed, the corresponding *δ*-function distribution *δ*(*θ* − *θ*_0_) will be mapped to the Gaussian distribution

$$\begin{array}{c} \rho \left(\theta \right)=N\left(\mu ,\sqrt{\nu }\right), \end{array}$$ (5)

with mean *μ* = *g* ^(*m*)^(*θ*_0_) given by Eq (23) and variance *ν* given by Eq (24). In order to study the infinite time behavior of Eq (2) it can be useful to calculate steady state probability distributions for the population of neurons. To simplify the analysis, we will study Eq (2) as a series of stochastic maps applied to an initial phase density (c.f. [22, 33]),

$$\begin{array}{c} \rho \left(\theta ,t+m\tau \right)=P_{m\tau }\rho \left(\theta ,t\right), \end{array}$$ (6)

where *P*_*mτ*_ is the linear Frobenius Perron operator corresponding to evolution for the time *mτ*, and *ρ*(*θ*, *t*) is the probability distribution of phases at time *t*. We can approximate *P*_*mτ*_ by the matrix $P_{m\tau }\in R^{M\times M}$ by using eq (5) to determine each column of the matrix for a set of discretized phases, Δ*θ* = 1/*M*. In Fig 4 for instance, the map *g* ^(2)^ yields the stochastic matrix $P_{2\tau }$, shown in the panel on the right. The delta function distribution (arrow) is mapped to a Gaussian distribution (solid line). The matrix $P_{m\tau }$ has all positive entries, and since probability is conserved, the matrix is column stochastic (i.e. the columns of $P_{m\tau }$ sum to 1). For this class of matrices, the Perron-Frobenius theorem allows us to write [36, 37],

$$\begin{array}{c} \lim_{k\to \infty }P_{m\tau }^{k}=vw^{T}, \end{array}$$ (7)

where *v* and *w* are the right and left eigenvectors associated with the unique eigenvalue of 1, and normalized so that *w* ^*T*^ *v* = 1. Recalling that $P_{m\tau }$ is column stochastic, its left eigenvector associated with *λ* = 1 is **1** ^*T*^. Therefore, as the map is applied repeatedly, any initial distribution will approach a steady state distribution determined by *v*. We find that the existence of *m* fixed points of the underlying map *g* ^(*m*)^(*θ*) provide the basis for the clustered desynchronization seen in Fig 2, with a more formal main theoretical result given below.

### Main Theoretical Result

Consider the map *g* ^(*m*)^ with the following properties:

1. *g* ^(*m*)^ has exactly *m* stable fixed points corresponding to a period *m* orbit of *g* ^(1)^,
2. *g* ^(*m*)^ has *m* unstable fixed points and no center fixed points,
3. *g* ^(*m*)^ is monotonic.

Then for a given choice of *ϵ*_1_ ≪ 1, we may choose *ϵ* small enough in eq (2) so that the distribution of phases will asymptotically approach a state with *m* distinct clusters, each containing $1/m+O(\epsilon_{1})$ of the total probability density. A proof of this statement is given in the Methods Section. In this detailed proof, we find that desynchronization can still be guaranteed even when *g* ^(*m*)^ is not monotonic as long as a more general set of conditions is satisfied. Note that because Eq (2) does not contain any coupling terms, noise will drive the system to a uniform, desynchronized, distribution in the absence of DBS input. In the sections to follow, we give numerical and theoretical evidence that clustered desynchronization can emerge in a population of pathologically synchronized neurons when the DBS pulses overwhelm the terms responsible for the synchronization.

### Arnold Tongues and Lyapunov Exponents

**Fig 5:**
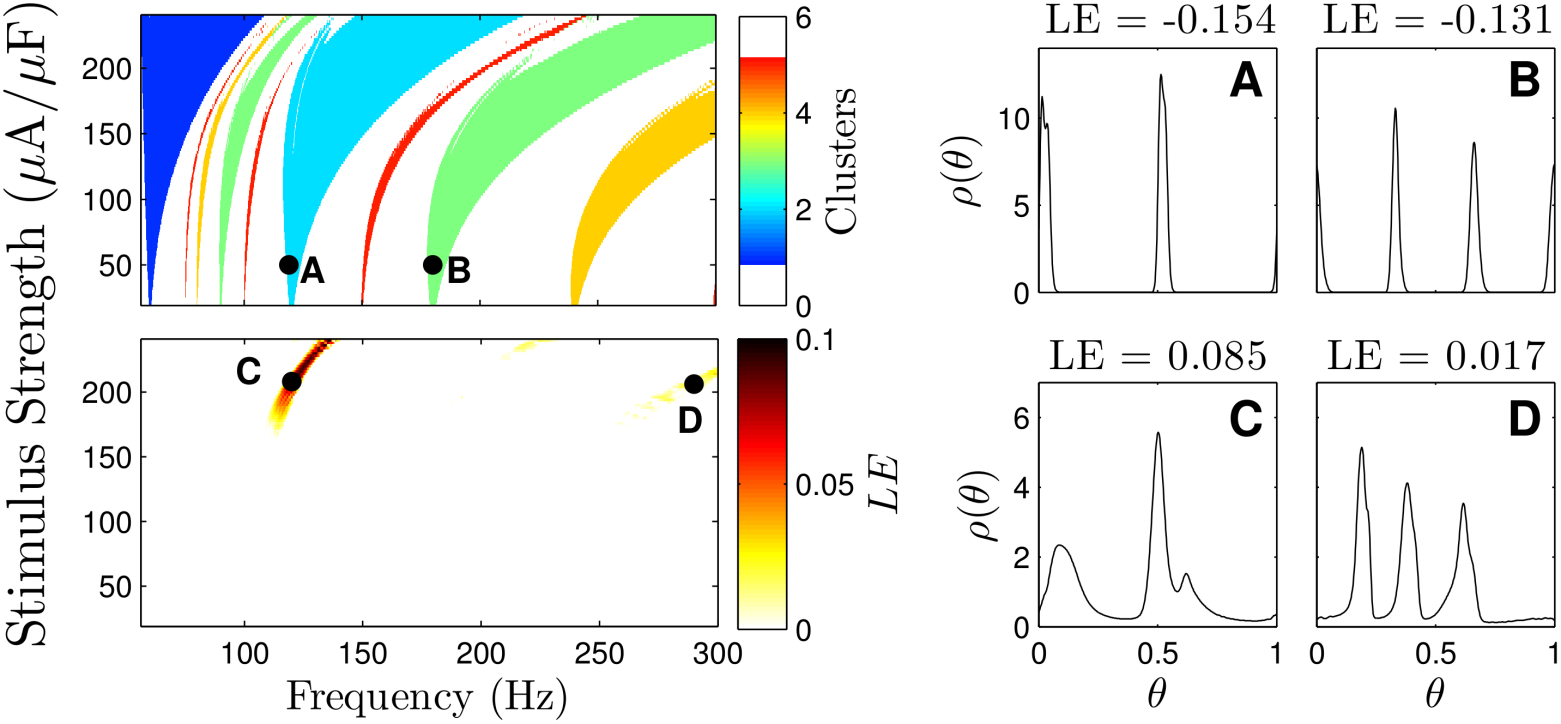
Clustered desynchronization and Lyapunov exponent calculations for thalamic neurons. The top-left panel shows regions of parameter space where clustered desynchronization is guaranteed for small enough noise. The bottom-left panel shows the regions associated with a positive average Lyapunov exponent (LE). Panels A-D give steady state probability distributions for stimulus parameters corresponding to black dots on the left.

Using the main theoretical result, we can calculate regions of parameter space where we expect clustered desynchronization. The top-left panel of Fig 5 gives regions of parameter space where clustering is expected, giving the appearance of Arnold tongues [35]. White regions in the graph represent regions where either clustering is not guaranteed, or where we expect more than five clusters. However, we do not include these regions in the figure because they only exist for very narrow regions of parameter space. At around 60 Hz, the natural unforced period of the neural population, exactly one cluster is guaranteed, corresponding to 1:1 locking (one DBS pulse per neural spike). This locking corresponds to a highly synchronous state, which we found when forcing the population at 63 Hz in Fig 2. For pulsing frequencies between 80 and 120 Hz, we see prominent tongues corresponding to states with three, four and five clusters, which correspond to the states in Fig 2 where we force at 83 Hz and 94 Hz. A very wide tongue corresponding to 2:1 locking (two DBS pulses per neural spike) exists at frequencies ranging from 120 to 200 Hz, which is where DBS is often seen to be effective. Pulsing in this region manifests in the behavior seen with 120 Hz forcing in Fig 2.

To make comparisons with [22] we calculate the average Lyapunov exponent of the resulting steady state distributions using Eq (12). For Lyapunov exponents greater than zero (resp., less than zero), the pulsatile stimulus will, on average, desynchronize (resp., synchronize) neurons which are close in phase, and this has been proposed as an indicator of the overall desynchronization that might be observed in a population of neurons receiving periodic DBS pulses. The Lyapunov exponent is calculated for multiple parameter values for a system with a noise strength *ϵ* = 0.1. Results are given in the bottom-left panel of Fig 5. We note that results are not qualitatively different for different noise strengths. Compared with the Arnold tongues in the top-left panel, we find very narrow regions where the Lyapunov exponent is positive at relatively high stimulus strengths. The top-right panels show the steady state distribution for a population with pulses of strength *S* = 50*μ*A/*μ*F at a rate of 119 Hz (resp., 180 Hz) corresponding to a two (resp., three) cluster steady state. The bottom-right panels show the steady state distribution for a pulsing strength of *S* = 208*μ*A/*μ*F at 120 Hz and *S* = 206*μ*A/*μ*F at 290 Hz corresponding to regions with positive Lyapunov exponents. Even though the clustered states have very negative Lyapunov exponents, they show similar clustering behavior to the states with a positive Lyapunov exponent. However, the clustered desynchronization in the top-right panels can be accomplished using a significantly weaker stimulus and can be observed at a much wider range of pulsing parameters.

### Heterogeneous Pulsatile Inputs

Results thus far have focused on populations of neurons receiving homogeneous pulsatile inputs. However, it is well established that voltage fields vary significantly with distance from an external voltage probe [38]. In computational models such heterogeneity has been shown to create complicated patterns of phase locking that are dependent on the stimulation strength [39] and can improve methods designed to desynchronize large populations of neurons [14].

To understand the emergence of clustered synchronization when external inputs are different among neurons, we can modify the stochastic differential eq (2) as follows

$$\begin{array}{c} \dot{\theta_{i}}=\omega +f_{i}\left(\theta_{i}\right)\delta \left(\text{mod}\left(t,\tau \right)\right)+\epsilon \eta_{i}\left(t\right)Z\left(\theta_{i}\right)+O\left(\epsilon^{2}\right),\;i=1,\ldots ,N. \end{array}$$ (8)

Here, *f*_*i*_(*θ*) is calculated based on the pulsatile input to each neuron. For each neuron, we use Eq (7) to calculate its steady state probability distribution. The state of each neuron is independent of the others, so that the average of the individual distributions gives an overall probability distribution for the population.

**Fig 6:**
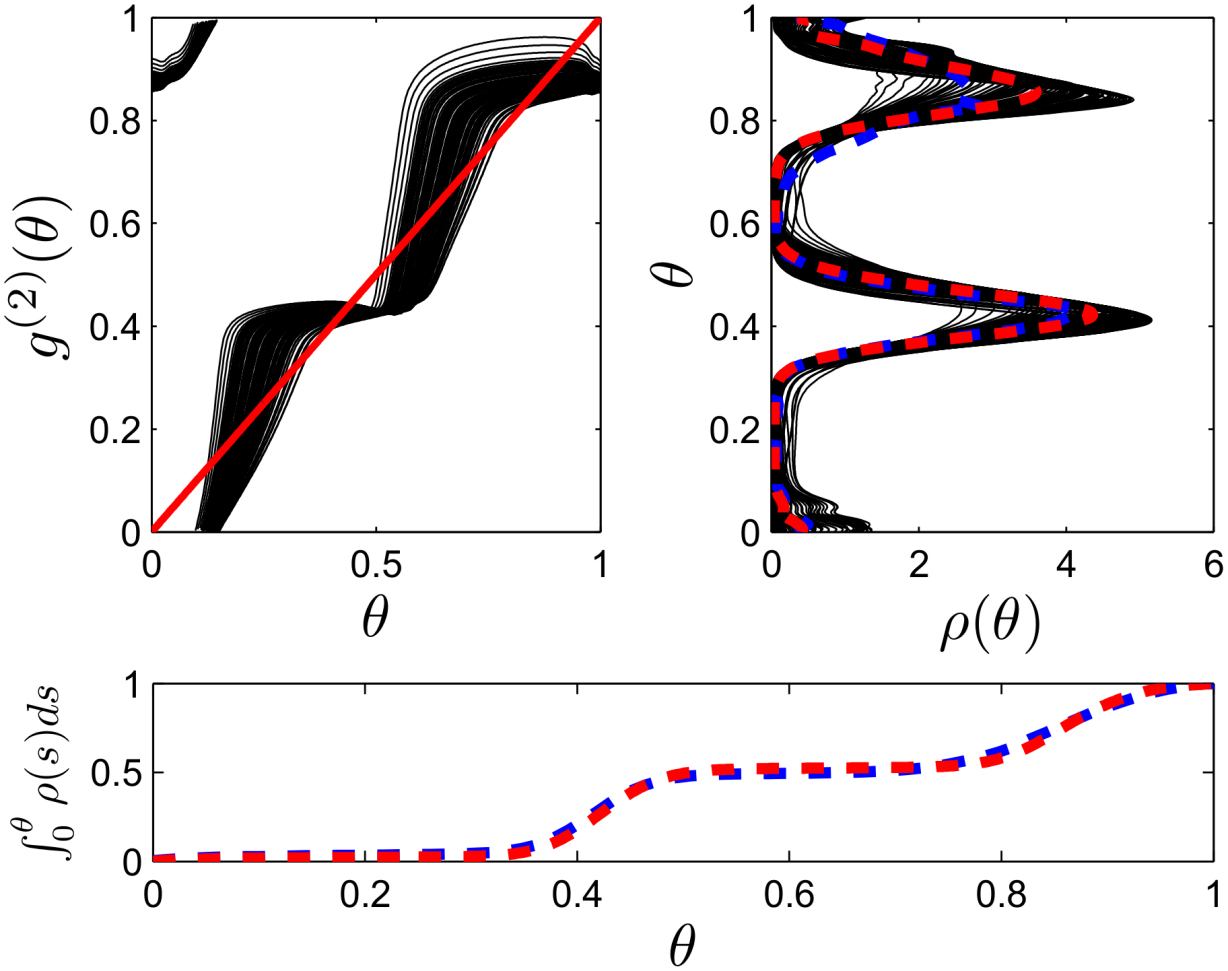
Heterogeneous pulsatile inputs. The top-left panel shows *g* ^(2)^(*θ*) (black lines) for DBS input at 140 Hz at multiple stimulation strengths with the identity line plotted in red for reference. The top-right panel shows the associated theoretical steady state probability distributions (black lines), average theoretical probability distribution (dashed red line), and computationally observed probability distributions (dashed blue line). The bottom panel shows the cumulative distributions for the average theoretical (red line) and computational (blue line) distributions.

As an illustrative example, we model 1000 neurons of the form Eq (1) receiving a charge balanced input of the same shape as in Fig 1 with *τ* = 1/(140 Hz) and *S* drawn from a normal distribution with mean 168 *μ*A/*μ*F and standard deviation 20, giving values of *S* between approximately 100 and 240. From the top-left panel of Fig 5, this range of stimulus parameters is mostly, but not completely, contained in a two cluster region. *g* ^(2)^(*θ*) is plotted in black in the top left panel of Fig 6 for a randomly chosen subset of these neurons with the identity line plotted in red for reference. The top-right panel shows each neuron’s steady state probability distribution (calculated from its associated stochastic matrix) in black for a noise strength of $\epsilon =\sqrt{0.4}$. While the main clustering results are guaranteed when the noise strength is small enough, we find that clustering can still occur at higher levels of noise. The steady state probability distribution in corresponding simulations of Eq (1) with heterogeneous pulsing strengths (blue dashed curve) agrees well with the theoretical probability density (red dashed curve) calculated from the average of each black curve in the top-right panel. The bottom panel shows corresponding cumulative distributions for the theoretical (red) and computationally observed (blue) probability densities highlighting that similar numbers of neurons are contained in each cluster.

Similar clustering results can be obtained for different heterogeneous stimulus parameters. For instance, from Fig 5, three-cluster behavior will emerge for pulsing frequencies of 200 Hz and stimulus strengths between approximately 90 and 170 *μ*A/*μ*F.

### Desynchronization of Neural Populations

Our main clustering results are for single population of neurons which do not explicitly take interactions between multiple populations of neurons into account, as is the case for the brain circuit responsible for Parkinsonian tremor. Here, we provide evidence that clustered desynchronization can still emerge when additional forcing terms are much smaller in magnitude than the external DBS pulses.

**Fig 7:**
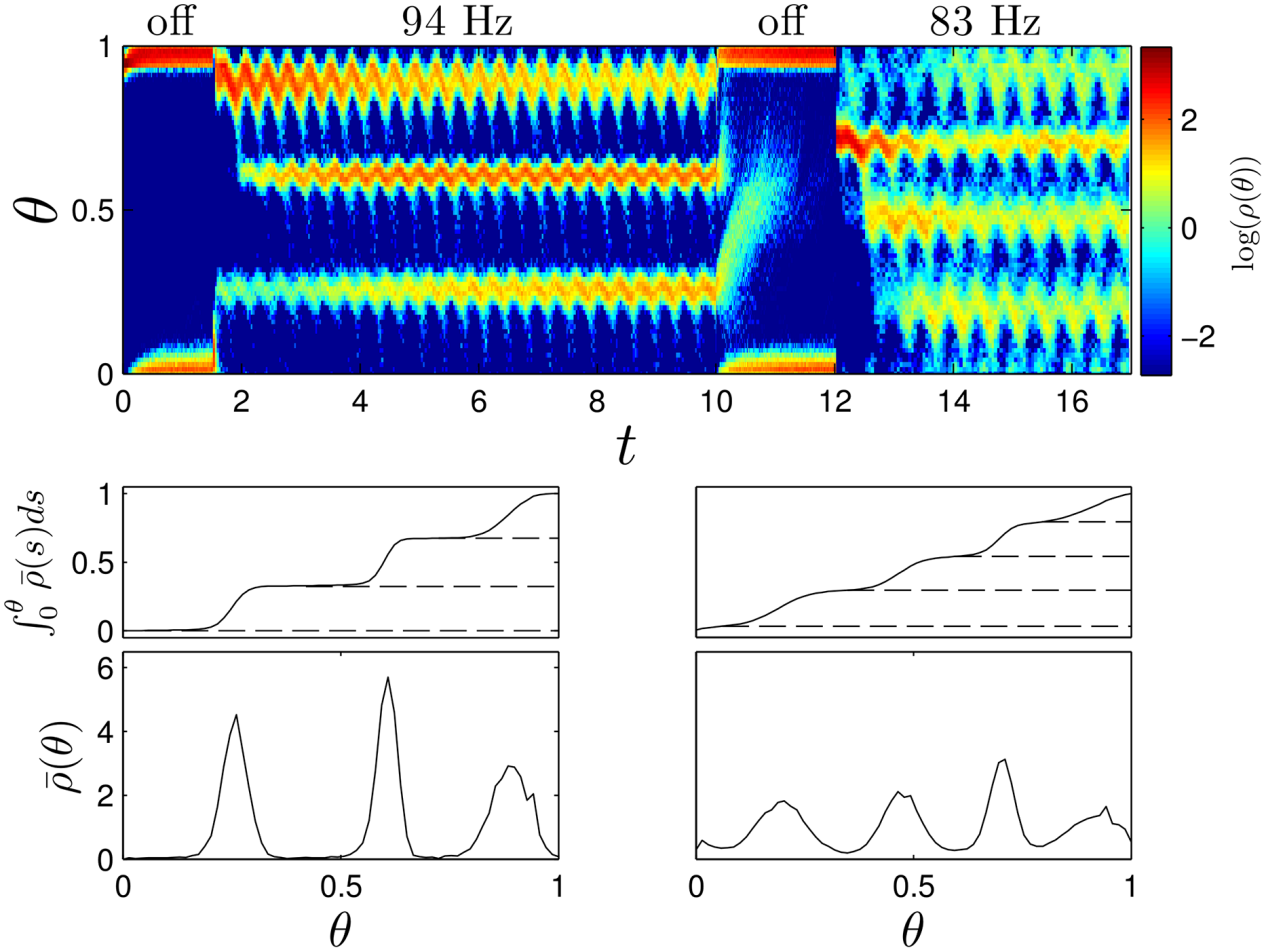
Simulations of (2) with additional sinusoidal forcing. In the top panel, snapshots of the probability distribution are taken immediately preceding every third and fourth pulse for the 83 and 94 Hz DBS pulsing, respectively. When there is no DBS pulsing, snapshots are taken at the sinusoidal forcing frequency. As averaging the term associated with the sinusoidal forcing would suggest, we observe desynchronization into three and four clusters for pulsing at 94 and 83 Hz pulsing, respectively. When the DBS pulsing is turned off at *t* = 8 seconds, the system quickly settles to a synchronized state. The bottom panels show the probability density averaged over multiple snapshots $\bar{\rho }\left(\theta \right)$ after the initial transient has decayed. Horizontal dashed lines denote troughs of the probability distributions. The probability contained between successive troughs is 0.32, 0.35, and 0.33 in the left panels, and 0.26, 0.26, 0.25, and 0.23 in the right panels.

Populations of coupled oscillators subject to common external forcing have been widely studied in the form of the forced Kuramoto model [40–42]. Synchronization can be observed when either external forcing or intrinsic coupling dominate the system dynamics. For intermediate coupling and external forcing strengths, a complicated bifurcation structure emerges and the macroscopic order parameter, describing the overall synchronization of the population, can oscillate. These behaviors have been observed in chemical oscillator systems [43] and have implications to externally forced biological rhythms such as circadian oscillations and neural oscillations [44]. We simulate Eq (1) with an additional external sinusoidal forcing frequency which could represent an aggregate input from a separate, unperturbed neural population. We note that this is not meant to represent a physiologically accurate model of DBS, but instead is meant to illustrate clustered desynchronization in the presence of a common periodic perturbation. Here, we take *u*(*t*) = 0.1 sin(*ω*_ext_ *t*) + *u*_DBS_(*t*), where *ω*_ext_ is chosen so that the frequency of oscillation is the same as the natural period of the unperturbed neurons (60 Hz) and *u*_DBS_(*t*) represents the common pulsatile input. For these simulations, *N* = 1000. As we show in the Methods Section we may write this system in an identical form as Eq (2), for which the main theoretical result still holds. For this particular example, clustering results are identical to those in the top left panel of Fig 5. Results are shown with a pulsing strength *S* = 110*μ*A/*μ*F and noise strength of $\epsilon =\sqrt{0.02}$ in Fig 7. We find that the presence of a sinusoidal external stimulus is sufficient to synchronize the network in the absence of DBS forcing. When the DBS is turned on at both 83 and 94 Hz, we see three and four cluster states, respectively, just as we observed in the simulation without external forcing. However, in this simulation, the mean phase of each cluster varies with the external sinusoidal stimulation. Note that 120 Hz stimulation in this network also leads to two cluster desynchronization but is not shown.

**Fig 8:**
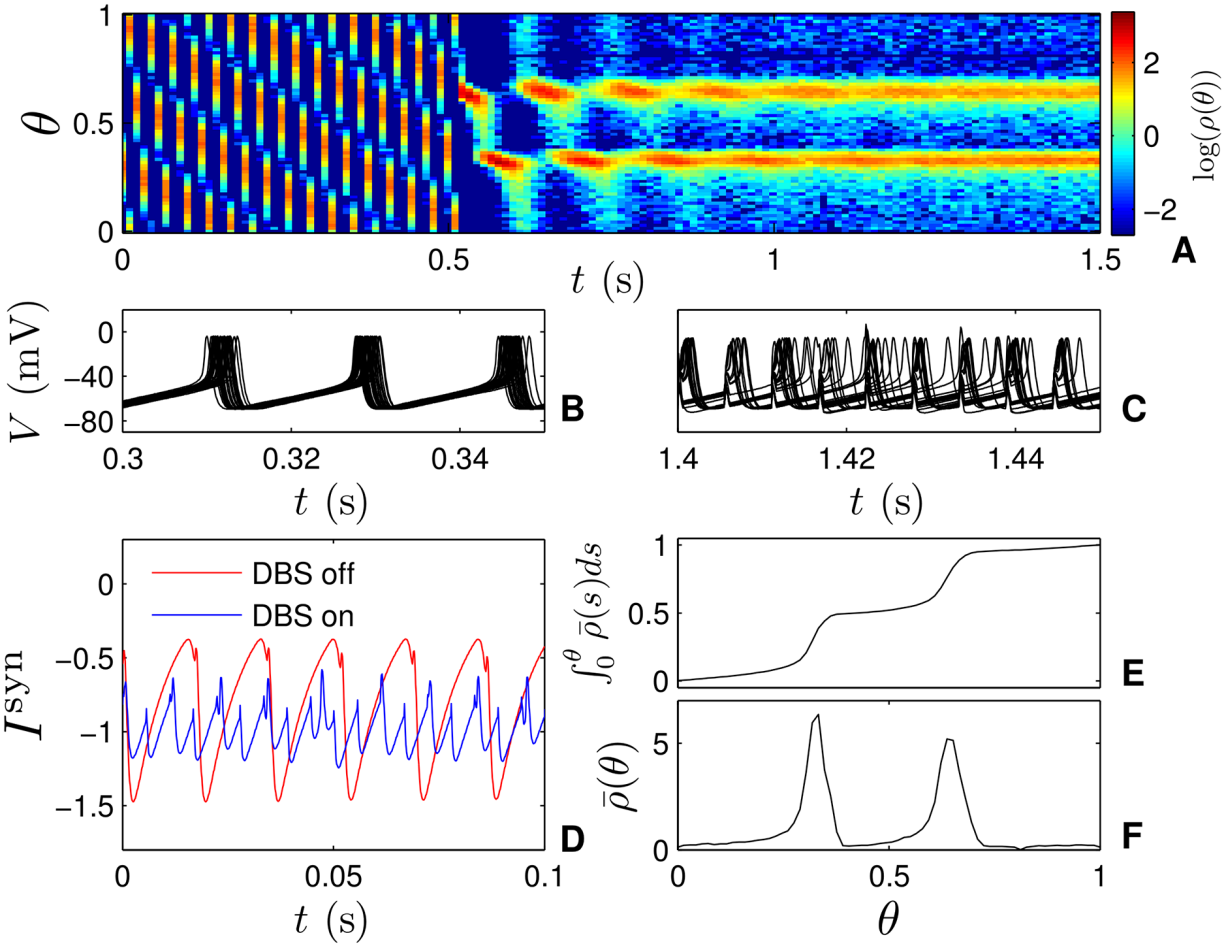
Simulations of (2) with synaptic coupling. Panels B and C show traces for 30 representative neurons from a synaptically coupled network without and with 180 Hz DBS pulsing, respectively. In Panel A, snapshots of the probability density are taken at 90 Hz. At *t* = 0.5 seconds, pulsatile stimulation is turned on. Panel D shows characteristic synaptic currents felt by a single neuron with and without DBS pulsing. Panel F shows the probability density $\bar{\rho }\left(\theta \right)$ averaged over multiple snapshots after initial transients have decayed when DBS pulsing is on and panel E gives the cumulative distribution. Here, we observe a similar clustering pattern, with nearly equal amounts of neurons in each cluster.

When neurons are synchronized through forcing that is not periodic, clustered desynchronization may still emerge when the DBS pulsing overwhelms the stimulation responsible for synchronization. As a second example, we model a network of neurons Eq (1) with an additional synaptic current, with each neuron’s transmembrane voltage dynamics taking the form $C\dot{V_{i}}=f_{V}\left(V_{i},h_{i},r_{i}\right)+I_{b}+u\left(t\right)+\epsilon \eta_{i}\left(t\right)+I_{i}^{\text{syn}}\left(t\right)$. Here,

$$\begin{array}{c} I_{i}^{\text{syn}}\left(t\right)=\frac{K}{N}\sum_{k=1}^{N}\left(V_{i}-V_{G}\right)s_{k}\left(t\right) \end{array}$$ (9)

where *K* determines the magnitude of the synaptic current, *V*_*G*_ is the reversal potential of a given neurotransmitter, and *s*_*k*_ an additional synaptic variable associated with neuron *k* that evolves according to (c.f. [30]) ${\dot{s}}_{k}=\alpha_{2}\left(1-s_{k}\right)\left(1/\left(1+\text{exp}\left(-\left(V_{k}-V_{T}\right)/\sigma_{T}\right)\right)\right)-\beta_{2}s_{k}$, where *α*_2_ = 2, *V*_*T*_ = -37, *σ*_*T*_ = 2, and *β*_2_ = 0.1. We simulate the resulting network with*V*_*g*_ = 60mV, *K* = 0.015 and a noise strength of $\epsilon =\sqrt{0.02}$; neurons form a single synchronized cluster in the absence of DBS input shown in panel B of Fig 8. starting at *t* = 0.5 ms, we apply 180 Hz stimulation with *S* = 200*μ*A/*μ*F, the pulsing quickly overwhelms the synchronizing influence of the coupling, and the population splits into two separate clusters as shown by the probability densities in Panels A and individual voltage traces in panel C. When DBS is applied, we see from the average probability distributions and cumulative distributions in panels F and E, respectively that there are nearly equal proportions of neurons in each cluster. Other computational modeling [29] has suggested that pulsatile DBS may help regulate neural firing patterns, and help alleviate strongly oscillatory synaptic inputs. Panel D shows a similar phenemenon, when DBS is on, high amplitude oscillations in synaptic current are replaced by oscillations with a higher frequency but smaller amplitude. The desynchronization results here can be observed for many choices of parameters provided the pulsatile stimulation is significantly stronger than the synchronizing stimulation and that clustering behavior is expected in the absence of coupling.

### Clustered Desynchronization in Type II Hodgkin-Huxley Neurons

Consider a two dimensional reduction of the classic Hodgkin-Huxley equations [45] which reproduce the essential dynamical behavior [46]:

$$\begin{array}{cc} C\dot{V_{i}} & =f_{V}^{H}\left(V_{i},n_{i}\right)+I_{b}+u\left(t\right)+\epsilon \eta_{i}\left(t\right),\\ \dot{n_{i}} & =f_{n}\left(V_{i},n_{i}\right),\;i=1,\ldots ,N. \end{array}$$ (10)

Here *V*_*i*_ and *n*_*i*_ represent the transmembrane voltage and gating variables, respectively. All functions and parameters are identical to those given in [47]. DBS pulses are represented by the external current *u*(*t*), which is given identically to each neuron, *η*_*i*_(*t*) is a white noise process, *C* = 1*μ*F/cm^2^ is the constant neural membrane capacitance, *I_b_* = 10*μ*A/*μ*F is a baseline current yielding a firing rate of 84.7 Hz in the absence of external perturbation, and *N* is the total number of neurons.

**Fig 9:**
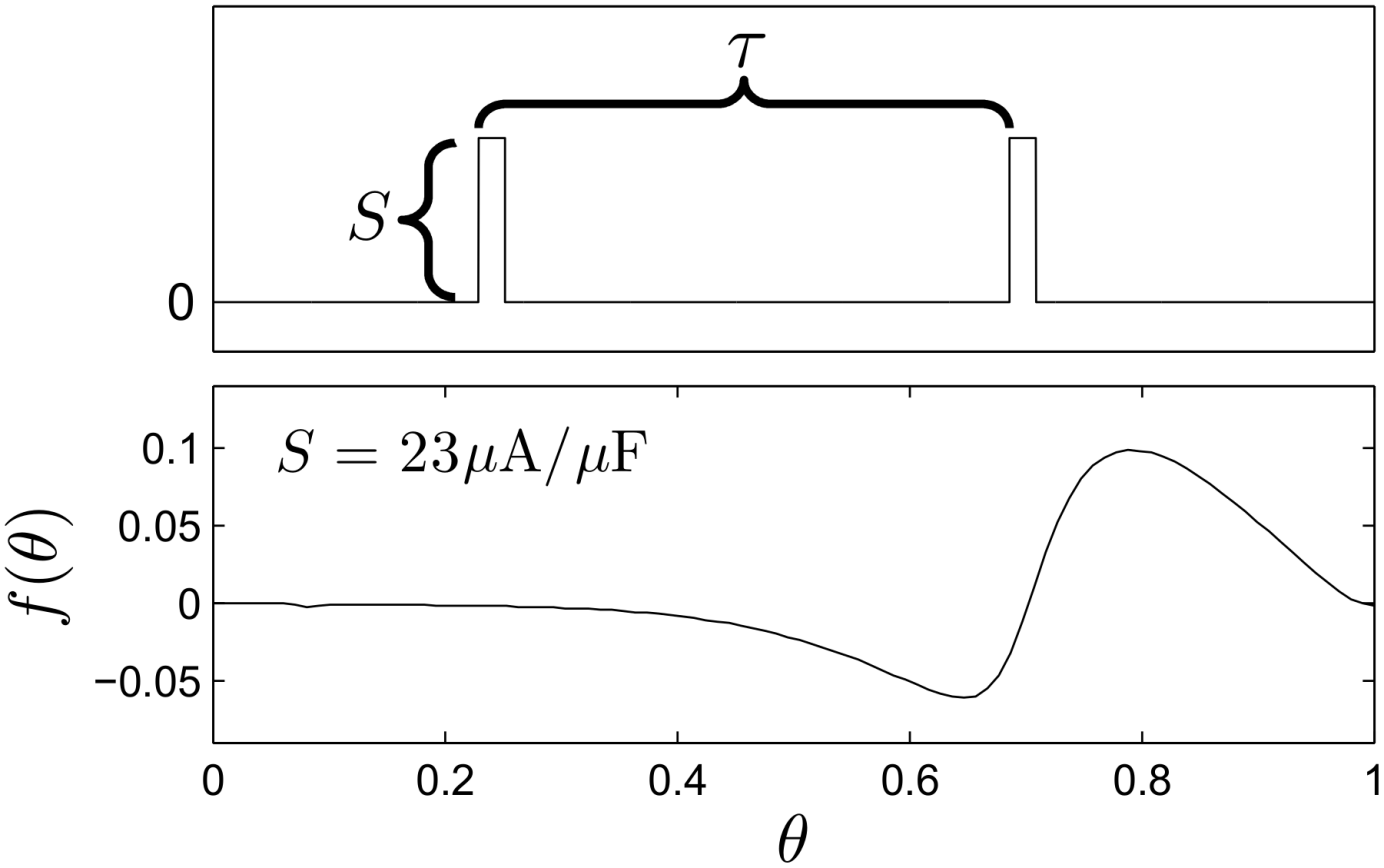
Monophasic DBS pulses. The top panel shows a pulsatile monophasic stimulus applied to the Hodgkin-Huxley neural network with frequency 1/*τ* and strength *S*. The bottom panel shows the relationship between pulse onset and the resulting change in phase.

Unlike the model for thalamic neurons used in the main text, the Hodgkin-Huxley neuron displays Type II phase response properties, i.e., a monophasic pulsatile input can act to either significantly increase or decrease the phase of the neuron. The top panel of Fig 9 shows an example monophasic stimulus which will be applied to the network Eq (10) at different strengths, *S* and periods *τ*. For this example, the pulse duration will be 100 *μs*, approximately, one percent of the neural firing rate.

**Fig 10:**
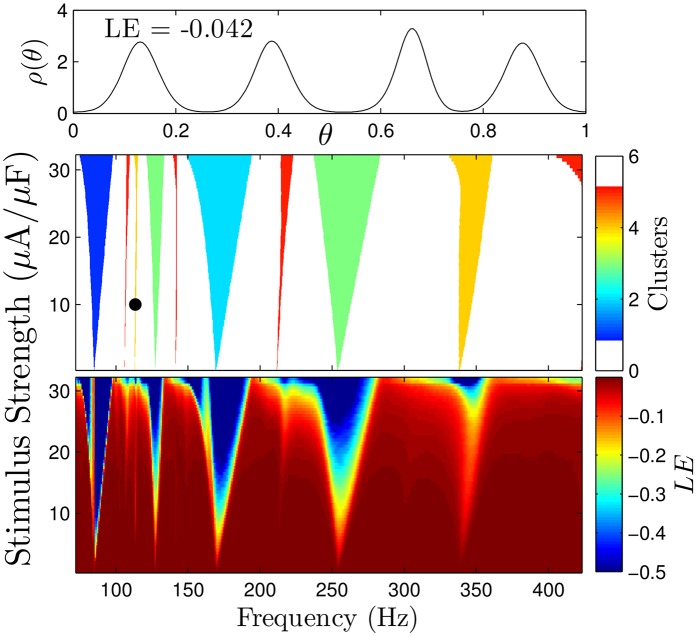
Clustering and Lyapunov exponent calculations for the Hodgkin-Huxley network. The middle panel gives regions of parameter space where clustered desynchronization is guaranteed to occur for small enough noise. The bottom panel shows the average Lyapunov exponent for the steady state distribution. The top panel shows the steady state probability distribution for stimulus parameters shown with black dots in the middle panel.

For this model, using our main theoretical results, we can also calculate regions of parameter space in which we expect to observe clustered desynchronization, with results shown in the middle panel of Fig 10. The Arnold tongues for clustering greater than five become quite narrow and are not included in this figure. We also calculate the average Lyapunov exponent for the steady state distribution using eq (12) from the main text for a noise strength of *ϵ* = 0.15, with results shown in the bottom panel. We note that unlike for the thalamic neurons, the Lyapunov exponent for the Hodgkin-Huxley network is never positive. We find that regions with the lowest Lyapunov exponents tend to correlate with regions where clustered desynchronization is guaranteed. Even though the Lyapunov exponent might be quite negative, the steady state distribution can still be relatively desynchronized if there are a large number of clusters, as evidenced by the four cluster state in the top panel.

**Fig 11:**
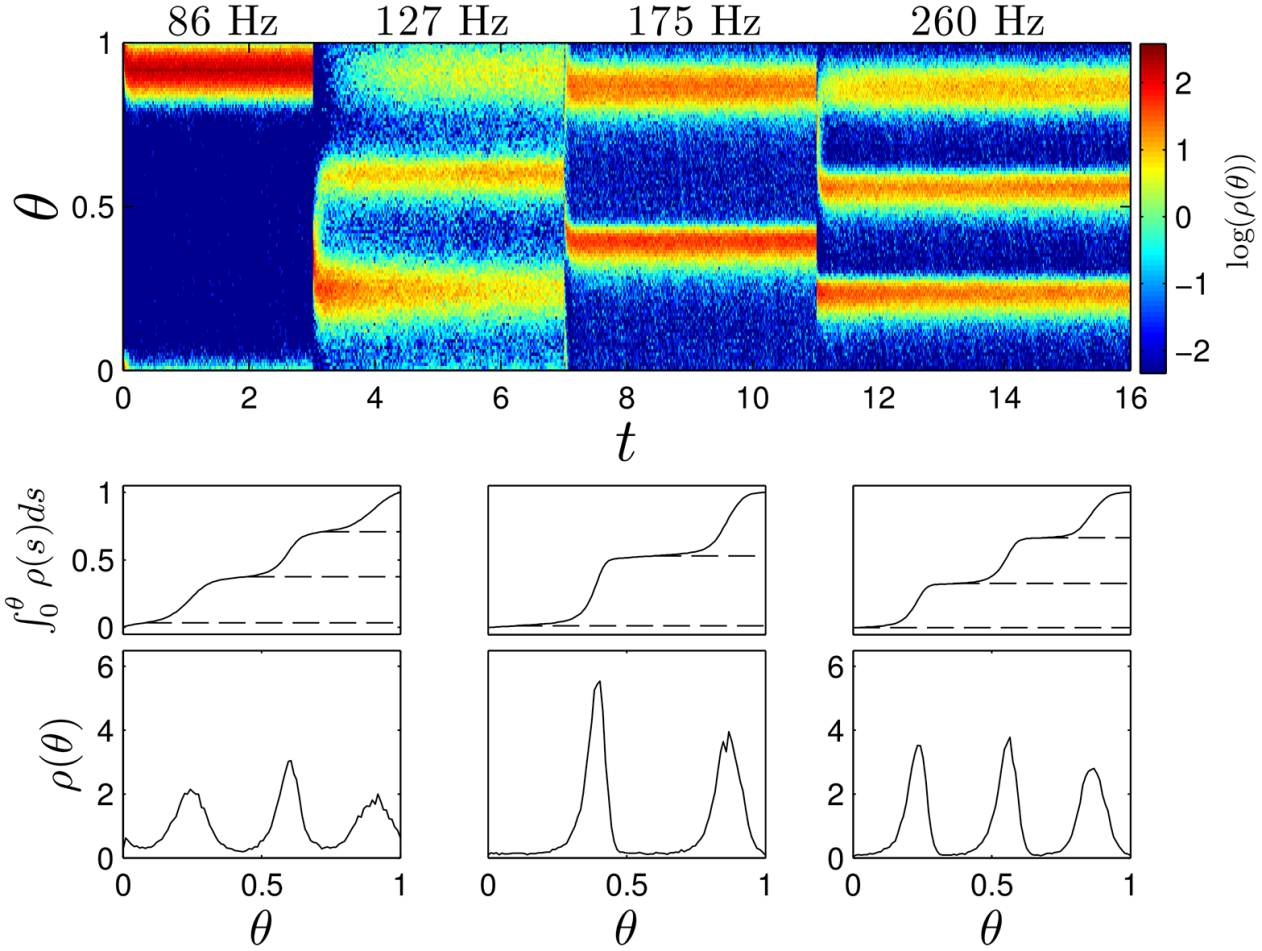
Clustered desynchronization in Type II reduced Hodgkin-Huxley neurons. The top panel shows snapshots of the probability distribution of phases *ρ*(*θ*) from of simulations of Eq (10). Snapshots are taken immediately preceding every pulse for the 86 Hz stimulation, and after every third, second, and third pulse for the 127, 175, and 260 Hz stimulation, respectively. From left to right, bottom panels show average probability distributions from the final fifty snapshots while stimulating at 127, 175, and 260 Hz stimulation, respectively. Horizontal dashed lines denote troughs of the probability distributions. The probability contained between successive troughs is 0.34, 0.33 and 0.33 in the left panels, 0.51, and 0.49 in the middle panels, and 0.33, 0.33, and 0.34 in the right panels.

Finally, we simulate Eq (10) with *N* = 1000 neurons with a pulse strength *S* = 10*μ*A/*μ*F and $\epsilon =\sqrt{0.3}$ for pulsing frequencies that are expected to yield clustered desynchronization determined from Fig 10. Results are shown in Fig 11. We find clustered states begin to form almost immediately, and in the bottom panel, after the system has approached the steady state distribution, each cluster contains an approximately identical proportion of the population.

## Discussion

While deep brain stimulation is an important treatment for patients with medically intractable Parkinson’s disease, its fundamental mechanisms remain unknown. Making matters more complicated, experimental studies have shown that the symptoms of Parkinson’s can be alleviated using strategies that seek to desynchronize a population of pathologically synchronized oscillators [15, 16], while other seemingly contradictory studies have shown that neurons have a tendency to time-lock to external high-frequency pulses [23–27], supporting the hypothesis that entrainment is necessary to replace the pathological neural activity in order to alleviate the symptoms of Parkinson’s disease. In this work we have have shown that these two phenomenon may be happening in concert: in the presence of a small amount of noise, high frequency pulsing at a wide range of frequencies is expected to split a larger population of neurons into subpopulations, each with a nearly equal proportion of the overall population. The number of subpopulations, and hence the level of desynchronization, is determined by phase locking relationships which can be found by analyzing the phase reduced system in the absence of noise. We note that other theoretical [12] and experimental [16][15] work has yielded control strategies that are specifically designed to split a pathologically synchronized neural population into distinct clusters. The theory presented in this paper suggests that clinical DBS may be accomplishing the same task with a single probe.

The conditions we have developed guarantee clustered desynchronization for small enough noise, but we do not give any *a priori* estimate of how small the noise needs to be so that distinct clusters can be observed. If the noise is too large, the clusters may start to merge into one another, particularly when there are a large number of clusters (see the bottom left panel of Fig 2). Even in this case, however, we still have discernible clusters, throughout which the overall population of neurons is spread relatively evenly. We also note that this theory does not give any estimates on the time it takes for the system to achieve its steady state population distribution, but this can be calculated for a specific population by examining the second smallest eigenvalue in magnitude, *λ*_2_, of a given stochastic matrix $P_{m\tau }$ (c.f. [33]). As *λ*_2_ becomes closer to 1, more iterations of the map $P_{m\tau }$ will be required for the transient dynamics to die out, and it will take longer for the system to approach the steady state distribution. In general, we find that for a given map, *λ*_2_ becomes closer to one as noise strength decreases, which is consistent with the notion that the average escape time between clusters will increase as the strength of the external noise decreases [48].

For the networks that we have investigated, regions of parameter space which are associated with either clustered desynchronization or positive Lyapunov exponents can display similar levels of desynchronization. However, numerics show that the regions with positive Lyapunov exponents are quite small and may be difficult to find without explicit calculation. In contrast, the regions of parameter space associated with clustered desynchronization are fairly large and are likely to be observed without knowledge of the system properties. If desynchronization of the overall population is an important mechanism of high-frequency DBS, doing so chaotically may be an overly restrictive objective if clustered desynchronization is sufficient to alleviate the motor symptoms of Parkinson’s disease.

This study is certainly not without limitations. For instance, the computational neurons considered in this study are based on simple, low-dimensional models of neural spiking behavior. However, we have developed the theory to understand the clustered desynchronization phenomenon in such a way that it can easily be extended to more complicated neural models with more physiologically detailed dynamics provided the neural phase response properties can be measured experimentally *in vivo*[49]. Furthermore, while we only considered homogeneous populations in this study, the phase response properties and natural frequencies of a living population of neurons will surely have a heterogeneous distribution. In this context, we could still show that clustered desynchronization is expected by applying the theory developed in this work to a family of neurons with different phase response properties and natural frequencies. The expected steady state population could then be obtained as a weighted average of the individual steady state distributions. Numerical results presented here apply to networks for which external DBS perturbations overwhelm the intrinsic coupling between neurons. In this work, we have not considered the complicated interplay between multiple populations of neurons which give rise to the symptoms of Parkinson’s disease; more detailed modelling studies would be required to determine the effect of clustered desynchronization on the overall network circuit. Others have studied synchrony and clustering behavior in coupled populations of neurons [50–52] and it is possible that our results could be extended to describe clustering for weaker pulsatile stimuli when coupling cannot be neglected.

Our results suggest that high-frequency external pulsing could have the effect of separating a neural population into equal subpopulations in the presence of noise. This viewpoint could help explain the frequency dependent nature of therapeutically effective DBS and could help merge competing hypotheses, as desynchronization and entrainment are not mutually exclusive when even small amounts of noise are present. If clustered desynchronization does provide a mechanism by which the motor symptoms associated with Parkinson’s disease can be mitigated, it could provide a useful control objective for designing better open-loop DBS stimuli in order to prolong battery life of the implantable device and to mitigate potential side effects of this therapy.

## Methods

### Average Lyapunov Exponents

To make comparisons with [22] we calculate the average Lyapunov exponent of the resulting steady state distributions, giving a sense of whether, on average, the orbits of the trajectories oscillators from Eq (2) are converging or diverging. For instance, let *ϕ* denote the phase difference between oscillators *θ*_1_ and *θ*_2_ which are close in phase, i.e. *ϕ*(*t*)≡|*θ*_1_(*t*) − *θ*_2_(*t*)|. Then from Eq (3), immediately after a DBS pulse occuring at time *τ*,

$$\begin{array}{cc} \phi (\tau^{+}) & =|f\left(\theta_{1}\left(\tau^{-}\right)\right)+\theta_{1}\left(\tau^{-}\right)-f\left(\theta_{2}\left(\tau^{-}\right)\right)-\theta_{2}\left(\tau^{-}\right)|,\\ & =|f\left(\theta_{2}\left(\tau^{-}\right)\right)+f^{\prime }\left(\theta_{2}\left(\tau^{-}\right)\right)\phi \left(\tau^{-}\right)+O{(\phi \left(\tau^{-}\right)}^{2})+\theta_{1}\left(\tau^{-}\right)-f\left(\theta_{2}\left(\tau^{-}\right)\right)-\theta_{2}\left(\tau^{-}\right)|,\\ & =\phi \left(\tau^{-}\right)|1+f^{\prime }\left(\theta_{2}\left(\tau^{-}\right)\right)|+O{(\phi \left(\tau^{-}\right)}^{2}), \end{array}$$ (11)

where ′ ≡ *d*/*dθ* and *θ*(*τ* ^−^) (resp. *θ*(*τ* ^+^)) denotes the limit of *θ*(*t*) as *t* approaches *τ* from below (resp. above). Note that in the second line, we have used a Taylor expansion of *f* about *θ*_2_ for small values of *ϕ*(*τ* ^−^). Therefore, the oscillators converge or diverge locally depending upon the derivative of *f*. For a population of neurons, the stochastic matrix $P_{\tau }$ for a given pulsing rate can be used to determine the steady state distribution ρ*(*θ*) before each pulse, with an average Lyapunov exponent taken to be (c.f. [22]):

$$\begin{array}{c} \text{LE}=\int_{0}^{1}\left(\rho^{*}\left(\theta \right)\log\left[1+f^{\prime }\left(\theta \right)\right]\right)d\theta . \end{array}$$ (12)

For LE > 0 (resp., LE < 0), the pulsatile stimulus will, on average, desynchronize (resp., synchronize) neurons which are close in phase, and this has been proposed as an indicator of the overall desynchronization that might be observed in a population of neurons receiving periodic DBS pulses.

### Expected Value and Variance of a Noisy Neuron with External Pulses

For a single neuron with a known initial phase *θ* evolving according to the stochastic differential Eq (2), we calculate the expected value and variance with a strategy that is similar to the one employed in [33]. We first asymptotically expand *θ*(*t*) in orders of *ϵ*,

$$\begin{array}{c} \theta \left(t\right)=\theta_{0}\left(t\right)+\epsilon \theta_{1}\left(t\right)+\ldots , \end{array}$$ (13)

with *θ*_0_(0) = *θ*(0), and *θ*_1_(0) = 0. Substituting Eq (13) into Eq (2) and taking *ω* = 1 for simplicity of notation, allows us to write

$${\dot{\theta }}_{0}=1+f\left(\theta_{0}\left(t\right)\right)\delta \left(\text{mod}\left(t,\tau \right)\right),$$ (14)

$${\dot{\theta }}_{1}=\eta \left(t\right)Z\left(\theta_{0}\left(t\right)\right)+f^{\prime }\left(\theta_{0}\left(t\right)\right)\theta_{1}\left(t\right)\delta \left(\text{mod}\left(t,\tau \right)\right).$$ (15)

Integrating eq (14) for a time *τ* yields,

$$\begin{array}{cc} \theta_{0}\left(\tau^{-}\right) & =\theta_{0}\left(0\right)+\tau ,\\ \theta_{0}\left(\tau^{+}\right) & =\theta_{0}\left(0\right)+f\left(\theta_{0}\left(0\right)+\tau \right)+\tau . \end{array}$$ (16)

This relationship can be used to write *θ*_0_ in terms of compositions of a map:

$$\begin{array}{c} \theta_{0}\left(t\right)=g^{\left(\lfloor \frac{t}{\tau }\rfloor \right)}\left(\theta_{0}\left(0\right)\right)+\text{mod}\left(t,\tau \right), \end{array}$$ (17)

where *g*(*s*) = *s* + *f*(*s* + *τ*) + *τ* and *g* ^(*n*)^ denotes the composition of *g* with itself *n* times. In Eqs (16) and (17), *θ*(*t*) and the arguments of *f* and *g* are always evaluated modulo 1.

We now focus our attention on *θ*_1_. Integrating eq (15) yields

$$\begin{array}{c} \theta_{1}\left(t\right)=\int_{0}^{t}\eta \left(s\right)Z\left(\theta_{0}\left(s\right)\right)ds+\sum_{m=1}^{\infty }f^{\prime }\left(\theta_{0}\left(m\tau^{-}\right)\right)\theta_{1}\left(m\tau^{-}\right)H\left(t-\tau m\right), \end{array}$$ (18)

where *H*(⋅) is the Heaviside step function. Note here that *θ*_1_(*mτ* ^−^) denotes the limit of *θ*_1_(*t*) as *t* approaches *mτ* from below. In the interval 0 ≤ *t* < *τ*, we note that $\theta_{1}\left(t\right)=\int_{0}^{t}\eta \left(s\right)Z\left(\theta_{0}\left(s\right)\right)ds$, so that $\theta_{1}\left(\tau^{-}\right)=\int_{0}^{\tau }\eta \left(s\right)Z\left(\theta_{0}\left(s\right)\right)ds$. With this in mind, we can rewrite Eq (18) as

$$\begin{array}{cc} \theta_{1}\left(t\right) & =\int_{0}^{t}\eta \left(s\right)Z\left(\theta_{0}\left(s\right)\right)ds+f^{\prime }\left(\theta_{0}\left(\tau^{-}\right)\right)\int_{0}^{\tau }\eta \left(s\right)Z\left(\theta_{0}\left(s\right)\right)ds\\ & =\left(1+f^{\prime }\left(\theta_{0}\left(\tau^{-}\right)\right)\right)\int_{0}^{\tau }\eta \left(s\right)Z\left(\theta_{0}\left(s\right)\right)ds+\int_{\tau }^{t}\eta \left(s\right)Z\left(\theta_{0}\left(s\right)\right)ds\;\text{for}\;\tau \leq t<2\tau . \end{array}$$ (19)

Likewise, using Eq (19) to determine *θ*_1_(2*τ* ^−^) allows us to write

$$\begin{array}{cc} \theta_{1}\left(t\right) & =\left[\left(1+f^{\prime }\left(\theta_{0}\left(\tau^{-}\right)\right)\right)+f^{\prime }\left(\theta_{0}\left(2\tau^{-}\right)\right)\left(1+f^{\prime }\left(\theta_{0}\left(\tau^{-}\right)\right)\right)\right]\int_{0}^{\tau }\eta \left(s\right)Z\left(\theta_{0}\left(s\right)\right)ds\\ & +\left(1+f^{\prime }\left(\theta_{0}\left(2\tau^{-}\right)\right)\right)\int_{\tau }^{2\tau }\eta \left(s\right)Z\left(\theta_{0}\left(s\right)\right)ds+\int_{2\tau }^{t}\eta \left(s\right)Z\left(\theta_{0}\left(s\right)\right)ds\;\text{for}\;2\tau \leq t<3\tau . \end{array}$$ (20)

This process can be repeated indefinitely to find

$$\begin{array}{cc} \theta_{1}\left(m\tau^{+}\right) & =X_{0}^{m}\int_{0}^{\tau }\eta \left(s\right)Z\left(\theta_{0}\left(s\right)\right)ds+X_{1}^{m}\int_{\tau }^{2\tau }\eta \left(s\right)Z\left(\theta_{0}\left(s\right)\right)ds\\ & +\cdots +X_{m-1}^{m}\int_{(m-1)\tau }^{m\tau }\eta \left(s\right)Z\left(\theta_{0}\left(s\right)\right)ds, \end{array}$$ (21)

where $X_{i}^{m}$ is defined recursively so that

$$\begin{array}{c} X_{i}^{m}=\left\{\begin{array}{cc} 0 & \text{if}\;m<i,\\ 1 & \text{if}\;m=i,\\ X_{i}^{m-1}+f^{\prime }\left(\theta_{0}\left(m\tau^{-}\right)\right)X_{i}^{m-1} & \text{if}\;m>i. \end{array}\right. \end{array}$$ (22)

Because Eq (21) is the sum of stochastic integrals which themselves are normally distributed random variables, *θ*_1_(*mτ* ^+^) will also be a normally distributed random variable [53]. Ultimately, we are interested in calculating the expected value and variance of a neuron starting at *θ*(0) after the application of *m* DBS inputs. Using the asymptotic expansion Eq (13), we can calculate the expected value of *θ*(*mτ* ^+^) as $E[\theta (m\tau^{+})]=E\left[\theta_{0}\left(m\tau^{+}\right)+\epsilon \theta_{1}\left(m\tau^{+}\right)+O\left(\epsilon^{2}\right)\right]$. Using the relations Eqs (17) and (21) and noting that the noise *η*(*s*) has a mean of zero, *E*[*θ*_1_(*mτ* ^+^)] = 0, and hence, the expected value, *μ*, is

$$\begin{array}{c} \mu \equiv E\left[\theta \left(m\tau^{+}\right)\right]=\theta_{0}\left(m\tau^{+}\right)=g^{\left(m\right)}\left(\theta \left(0\right)\right)+O\left(\epsilon^{2}\right). \end{array}$$ (23)

Again using Eqs (17) and (21), we can calculate the variance, *ν*, of *θ*(*mτ* ^+^) to leading order *ϵ* ^2^:

$$\begin{array}{cc} \nu & \equiv E\left[(\theta \left(m\tau^{+}\right)-E[\theta {\left(m\tau^{+})]\right)}^{2}\right]\\ & =E[\left(\epsilon \theta_{1}{\left(m\tau^{+})\right)}^{2}\right]\\ & =\epsilon^{2}\left[{\left(X_{0}^{m}\right)}^{2}\int_{0}^{\tau }\left[Z^{2}\left(\theta_{0}\left(s\right)\right)\right]ds+{\left(X_{1}^{m}\right)}^{2}\int_{\tau }^{2\tau }\left[Z^{2}\left(\theta_{0}\left(s\right)\right)\right]ds+\cdots +{\left(X_{m-1}^{m}\right)}^{2}\int_{(m-1)\tau }^{m\tau }\left[Z^{2}\left(\theta_{0}\left(s\right)\right)\right]ds\right]. \end{array}$$ (24)

Note that in the last line, when squaring *θ*_1_(*mτ* ^+^), the fact that any noise processes that do not overlap in time are uncorrelated allows us to eliminate terms nonidentical noise processes. Also note that for identical white noise processes, *E*[*η*(*s*)*η*(*s*)] = *δ*(0).

### Proof of Clustered Desynchronization in the Limit of Small Noise

**Fig 12:**
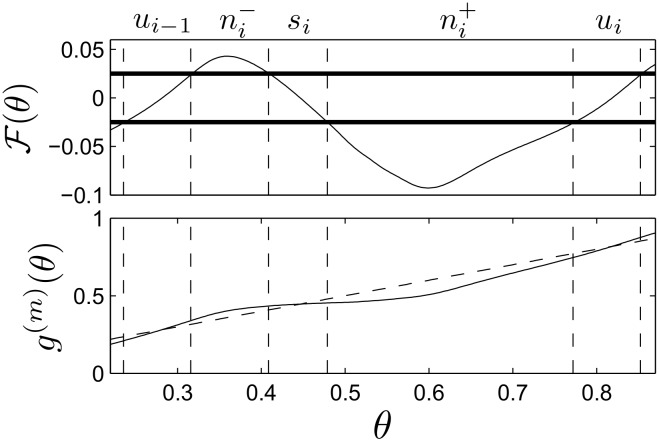
Example partitioning near a stable fixed point. In the top panel, horizontal bars represent a particular choice of *α*. Vertical lines separate the resulting regions. The bottom panel shows the corresponding regions for *g* ^(*m*)^(*θ*) (solid line). For reference, the diagonal dotted identity line is also shown.

**Fig 13:**
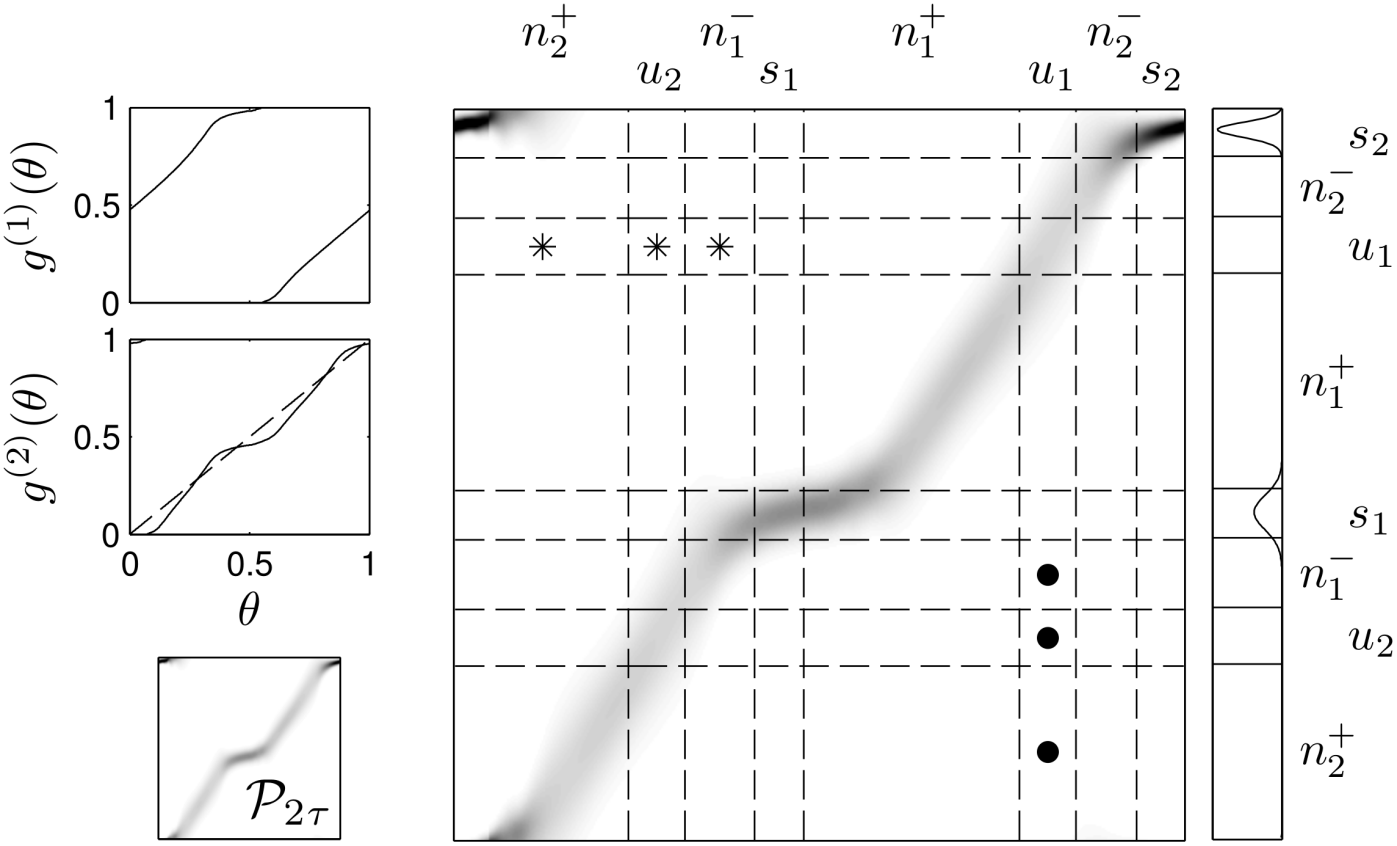
Example partitioning of the matrix to test conditions 3a-c. The left panels show an example of *g* ^(1)^ and *g* ^(2)^ and $P_{2\tau }$. Note that *g* ^(2)^ has two stable and two unstable fixed points. The partition of $P_{2\tau }$ shown in the right panel with dashed lines can be determined from a given choice of *α*. We note that the matrix has been flipped in the vertical direction to emphasize the correspondence between $P_{2\tau }$ and *g* ^(2)^(*θ*) so that in this visual example, matrix multiplication would not be performed in the usual way. From left to right, the submatrices denoted with asterisks are defined to be $P_{2\tau }^{n_{2}^{+}\to u_{1}}$, $P_{2\tau }^{u_{2}\to u_{1}}$, and $P_{2\tau }^{n_{1}^{-}\to u_{1}}$. From top to bottom, the regions submatrices denoted with dots are defined to be $P_{m\tau }^{u_{1}\to n_{1}^{-}}$, $P_{m\tau }^{u_{1}\to u_{2}}$, and $P_{m\tau }^{u_{1}\to n_{2}^{+}}$. The steady state distribution calculated as the right eigenvector of $P_{2\tau }$ associated with *λ* = 1 is shown to the right of $P_{2\tau }$, along with the associated partition. Note that because the conditions guaranteeing clustered desynchronization are satisfied, by taking *ϵ* small enough, the difference between the amount of the steady state population found in *s*_1_ and *s*_2_ can be made arbitrarily small.

Suppose that conditions 1 and 2 from our main theoretical results are satisfied for *g* ^(*m*)^ with *m* stable fixed points. Consider the corresponding stochastic matrix $P_{m\tau }$. Denote the stable and unstable fixed points as *θ*_*s*_*i*__ and *θ*_*u*_*i*__, respectively. To begin, it will be convenient to define

$$\begin{array}{c} g^{\left(m\right)}\left(\theta \right)=\theta +F\left(\theta \right) \end{array}$$ (25)

so that $F\left(\theta \right)\equiv g^{\left(m\right)}\left(\theta \right)-\theta$. With this definition, F(θ)=0 corresponds to the fixed points of the map *g* ^(*m*)^(*θ*). We subdivide *θ* ∈ [0, 1) into into 4*m* disjoint subregions with the following procedure: Choose *α* > 0 and define the region *s*_*i*_ near each stable fixed point so that $s_{i}=\left[\theta_{s_{i}}-\delta_{s_{i}}^{-},\theta_{s_{i}}+\delta_{s_{i}}^{+}\right]$, where $\alpha =F\left(\theta_{s_{i}}-\delta_{s_{i}}^{-}\right)=-F\left(\theta_{s_{i}}+\delta_{s_{i}}^{+}\right)$. Likewise, define the region *u*_*i*_ near each unstable fixed point so that $u_{i}=\left[\theta_{u_{i}}-\delta_{u_{i}}^{-},\theta_{u_{i}}+\delta_{u_{i}}^{+}\right]$, where $\alpha =-F\left(\theta_{u_{i}}-\delta_{u_{i}}^{-}\right)=F\left(\theta_{u_{i}}+\delta_{u_{i}}^{+}\right)$. Define the remaining regions $n_{i}^{+}=\left(\theta_{s_{i}}+\delta_{s_{i}}^{+},\theta_{u_{i}}-\delta_{u_{i}}^{-}\right)$ and $n_{i}^{-}=\left(\theta_{u_{i-1}}+\delta_{u_{i-1}}^{+},\theta_{s_{i}}-\delta_{s_{i}}^{-}\right)$. See Figs 12 and 13 for an example of how the matrix $P_{m\tau }$ is partitioned into submatrices according to this procedure.

**Fig 14:**
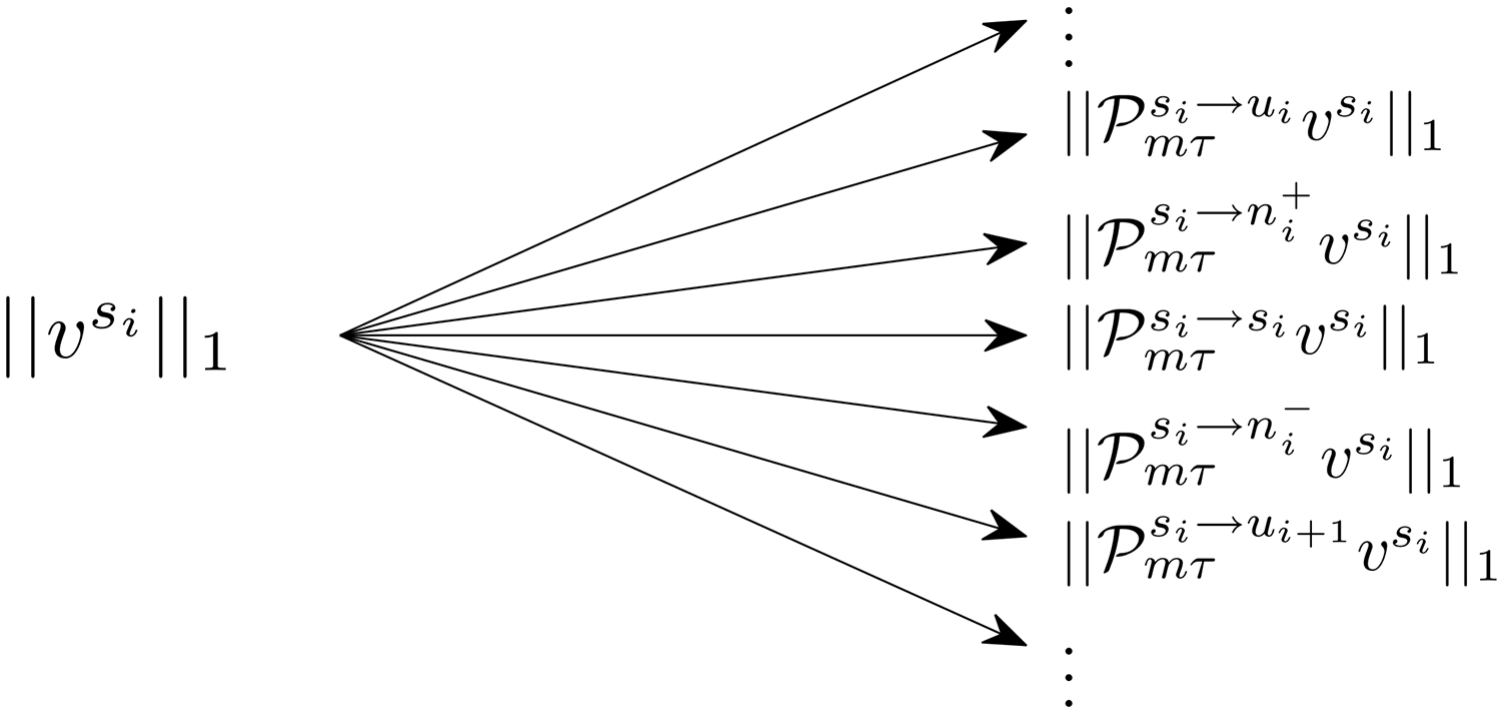
Mapping probability between regions. In the limit as time approaches infinity, the probability that a neuron will be found in the region *s*_*a*_ for $a=s_{i},u_{i},n_{i}^{+},n_{i}^{-}$ is given by $\left|\right|v^{s_{a}}{\left|\right|}_{1}$. After the time *mτ* has elapsed, the probability that a randomly chosen neuron started in *s*_*a*_ and was mapped to *s*_*b*_ for $b=s_{i},u_{i},n_{i}^{+},n_{i}^{-}$ is given by $\left|\right|P_{m\tau }^{s_{a}\to s_{b}}v^{s_{a}}{\left|\right|}_{1}$. Based on these relations, the above diagram gives an example characterizing how probability initially found in the region *s*_*i*_ is mapped to all other regions after the time *mτ* has elapsed.

In the analysis to follow we will show that the steady state probability distribution $v\equiv \lim_{k\to \infty }P_{m\tau }^{k}\rho \left(\theta ,0\right)$ exists and is invariant to *ρ*(*θ*, 0). We define subregions of $v^{a},\;a=s_{i},u_{i},n_{i}^{+},n_{i}^{-}$ to represent the subset of *v* contained in the region *a*. We have carefully defined the matrix partition in Fig 13 so that, for instance, $\left|\right|P_{m\tau }^{s_{i}\to n_{i}^{+}}v^{s_{i}}{\left|\right|}_{1}$ corresponds to the amount of probability that is mapped from the region *s*_*i*_ into to the region $n_{i}^{+}$ when $P_{m\tau }$ is applied to *v*. This relation results because all entries of P and *v* are positive. See Fig 14 for a visual representation of this probability mapping process which is central to the proof to follow.

Suppose that there exists *α* for the resulting partition such that,

3a for all $\theta \in n_{i}^{+}$ (resp., $n_{i}^{-}$), $g^{\left(m\right)}\left(\theta \right)\in n_{i}^{+}$ (resp., $n_{i}^{-}$) ∪*s*_*i*_
3b for all *θ* ∈ *s*_*i*_, $g^{\left(m\right)}\left(\theta \right)\in {\overset{˚}{s}}_{i}$
3c for all *θ* ∈ *u*_*i*_, $\frac{d}{d\theta }g^{\left(m\right)}{|}_{\theta }>1$ and $g^{\left(m\right)}\left(\theta \right)\notin {\bar{u}}_{j}$ for *i* ≠ *j*

Note here that for a given set *p*, $\overset{˚}{p}$ denotes its interior, and $\bar{p}$ denotes its closure. Furthermore, we will also assume, without loss of generality, that in the absence of noise, upon successive iterations of the map *g* ^(1)^ the period *m* orbit is *θ*_*s*_1__ → *θ*_*s*_2__ → ⋯ → *θ*_*s*_*m*__ → *θ*_*s*_1__. Let *γ* = mod(*i*, *m*) + 1. Suppose then that

3d for all *θ* ∈ *s*_*i*_, *g* ^(1)^(*θ*)∈ the interior of $n_{\gamma }^{-}\cup s_{\gamma }\cup n_{\gamma }^{+}$

Then for any choice of *ϵ*_1_ ≪ 1, we may choose *ϵ* (the noise strength) small enough in eq (2), so that as time approaches infinity, regardless of initial conditions, the population will be split into *m* distinct clusters, and to leading order in *ϵ*_1_, each cluster will contain an equal portion of the population. We note that if conditions 1 and 2 from our main theoretical result are satisfied and *g* ^(*m*)^ is monotonic, then we will be guaranteed to be able to choose and *α* so that the remaining conditions 3a-d are satisfied.

In order to prove the main theoretical result presented earlier, we will first show that for *ϵ* small enough, as time tends toward infinity, the probability of finding an oscillator far from any of the stable fixed points is $O(\epsilon_{1})$. We will then show that as time approaches infinity, the chance of finding a randomly chosen oscillator near any of the stable fixed points is identical to leading order *ϵ*_1_.

Throughout this proof, we are interested in the unique steady state solution which solves $v=P_{\tau }v$. We note that for any positive integer *k*, $P_{k\tau }=P_{\tau }^{k}$ so that $v=P_{k\tau }v$, i.e. the unique steady state solution of $P_{k\tau }$ is the same for any choice of *k*. Using Eqs (23) and (24), we will assume that *ϵ* is taken small enough so that errors in the approximations of all necessary stochastic matrices $P_{k\tau }$ are negligible.

#### Bounding near unstable fixed points

To begin, for the stochastic matrix $P_{m\tau }$, we intend to bound the row sums of the submatrix $P_{m\tau }^{u_{i}\to u_{i}}$, the portion of the matrix $P_{m\tau }$ which maps *u*_*i*_ back to *u*_*i*_ (see Fig 13 for an example of this notation). Let *μ*_*θ*_ and *σ*_*θ*_ correspond to the expected value and standard deviation of any oscillator with initial condition *θ*, respectively. Let *θ*_*L*_ and *θ*_*R*_ correspond to the left and right boundaries of *u*_*i*_. We assume that phase space is partitioned into equal bins of length $\Delta \theta =1/M=O(\epsilon_{1})$, where $P_{m\tau }\in R^{M\times M}$. For row *j* of $P_{m\tau }^{u_{i}\to u_{i}}$ corresponding to *θ*_*j*_, its row sum *R*_*j*_ can be calculated as

$$\begin{array}{cc} R_{j} & =\left[N\left(\mu_{\theta_{L}}-\theta_{j},\sigma_{\theta_{L}}\right)+N\left(\mu_{\theta_{L}+\Delta \theta }-\theta_{j},\sigma_{\theta_{L}+\Delta \theta }\right)+\ldots \right.\\ & +\left.N\left(\mu_{\theta_{R}-\Delta \theta }-\theta_{j},\sigma_{\theta_{R}-\Delta \theta }\right)+N\left(\mu_{\theta_{R}}-\theta_{j},\sigma_{\theta_{R}}\right)\right]\Delta \theta , \end{array}$$ (26)

where $N\left(\theta ,\sigma \right)=\frac{1}{\sigma \sqrt{2\pi }}\exp\left(\frac{-\theta^{2}}{2\sigma^{2}}\right)$ is the equation for a Gaussian curve. As we showed in Eq (23), *μ*_*θ*_ = *g* ^(*m*)^(*θ*). Let $\theta_{E}=\text{arg}\min_{\theta \in [\theta_{L},\theta_{R}]}\left(\right|\mu_{\theta }-\theta_{j}\left|\right)$ correspond to the closest to *θ*_*j*_ any oscillator in *u*_*i*_ is expected to map to. Taylor expanding around *θ*_*E*_ yields

$$\begin{array}{cc} g^{\left(m\right)}\left(\theta \right) & =g^{\left(m\right)}\left(\theta_{E}\right)+g^{{\left(m\right)}^{\prime }}\left(\theta_{E}\right)\left(\theta -\theta_{E}\right)+O\left({\left(\theta -\theta_{E}\right)}^{2}\right)\\ & =\mu_{\theta_{E}}+g^{{\left(m\right)}^{\prime }}\left(\theta_{E}\right)\left(\theta -\theta_{E}\right)+O\left({\left(\theta -\theta_{E}\right)}^{2}\right). \end{array}$$ (27)

Similarly, we Taylor expand *σ*_*θ*_ around *θ*_*E*_ as

$$\begin{array}{c} \sigma_{\theta }=\sigma_{\theta_{E}}+O\left(\theta -\theta_{E}\right). \end{array}$$ (28)

Substituting Eqs (27) and (28) into Eq (26) yields,

$$\begin{array}{cc} R_{j} & =\left[\cdots +N(\mu_{\theta_{E}}-2\Delta \theta g^{{\left(m\right)}^{\prime }}\left(\theta_{E}\right)+O\left({\left(2\Delta \theta \right)}^{2}\right)-\theta_{j},\sigma_{\theta_{E}}+O\left(2\Delta \theta \right))\right.\\ & +N(\mu_{\theta_{E}}-\Delta \theta g^{{\left(m\right)}^{\prime }}\left(\theta_{E}\right)+O\left({\left(\Delta \theta \right)}^{2}\right)-\theta_{j},\sigma_{\theta_{E}}+O\left(\Delta \theta \right))\\ & +N(\mu_{\theta_{E}}-\theta_{j},\sigma_{\theta_{E}})\\ & +N(\mu_{\theta_{E}}+\Delta \theta g^{{\left(m\right)}^{\prime }}\left(\theta_{E}\right)+O\left({\left(\Delta \theta \right)}^{2}\right)-\theta_{j},\sigma_{\theta_{E}}+O\left(\Delta \theta \right))\\ & +\left.N(\mu_{\theta_{E}}+2\Delta \theta g^{{\left(m\right)}^{\prime }}\left(\theta_{E}\right)+O\left({\left(2\Delta \theta \right)}^{2}\right)-\theta_{j},\sigma_{\theta_{E}}+O\left(2\Delta \theta \right))+\cdots \right]\Delta \theta . \end{array}$$ (29)

For a given choice of *ϵ*_1_ ≪ 1, we may choose *ϵ* small enough yielding a standard deviation *σ*_*θ*_*E*__ small enough so that Eq (29) can be approximated to leading order *ϵ*_1_ with an arbitrarily small number of terms. For the remaining terms, notice that $\frac{\partial }{\partial \theta }q\left(\theta ,\sigma \right)=\frac{-\theta }{\sigma^{3}\sqrt{2\pi }}\exp\left(\frac{-\theta^{2}}{2\sigma^{2}}\right)$ and that $\frac{\partial }{\partial \sigma }q\left(\theta ,\sigma \right)=\frac{1}{\sqrt{2\pi }\sigma^{2}}\exp\left(\frac{-\theta^{2}}{2\sigma^{2}}\right)[\theta^{2}/\sigma^{2}-1]$. Therefore, if we choose *ϵ* small enough, *σ*_*θ*_*E*__ will be small enough so that the remaining Taylor expanded terms contribute at most $O(\epsilon_{1})$ error. When we do this we may rewrite eq (29) as

$$\begin{array}{cc} R_{j}= & \left[\cdots +N(-2\Delta \theta g^{{\left(m\right)}^{\prime }}\left(\theta_{E}\right),\sigma_{\theta_{E}})\right.\\ & +N(-\Delta \theta g^{{\left(m\right)}^{\prime }}\left(\theta_{E}\right),\sigma_{\theta_{E}})\\ & +N(0,\sigma_{\theta_{E}})\\ & +N(\Delta \theta g^{{\left(m\right)}^{\prime }}\left(\theta_{E}\right),\sigma_{\theta_{E}})\\ & +\left.N\left(2\Delta \theta g^{{\left(m\right)}^{\prime }}\left(\theta_{E}\right),\sigma_{\theta_{E}}\right)+\cdots +O\left(\epsilon_{1}\right)\right]\Delta \theta . \end{array}$$ (30)

Recall that *μ*(*θ*_*E*_) − *θ*_*j*_ is $O(\epsilon_{1})$ so that it can be lumped with the other $O(\epsilon_{1})$ terms upon Taylor expansion. Notice that Eq (30) can be written as a Riemann sum approximation to

$$\begin{array}{cc} R_{j} & =\int_{\theta_{L}}^{\theta_{R}}N\left(\left(\theta -\theta_{j}\right)g^{{\left(m\right)}^{\prime }}\left(\theta_{E}\right),\sigma_{\theta_{E}}\right)d\theta +O\left(\epsilon_{1}\right)\\ & =\int_{\theta_{L}}^{\theta_{R}}\frac{1}{\sigma_{\theta_{E}}\sqrt{2\pi }}\exp\left(\frac{-{\left(\left(g^{{\left(m\right)}^{\prime }}\left(\theta_{E}\right)\right)\left(\theta -\theta_{j}\right)\right)}^{2}}{2\sigma_{\theta_{E}}^{2}}\right)d\theta +O\left(\epsilon_{1}\right). \end{array}$$ (31)

The integral in Eq (31) is less than one because *g*′^(*m*)^(*θ*_*k*_)>1 so that it falls off more quickly than a Gaussian distribution. Therefore, if we choose *Δθ* and *ϵ*_1_ small enough, *R*_*j*_ < 1, which implies $\left|\right|P_{m\tau }^{u_{i}\to u_{i}}{\left|\right|}_{\infty }<1$.

Because the column sums of the stochastic matrix $P_{m\tau }$ are all equal to one, and each entry is positive, we may use Gershgorin disks to show all eigenvalues are less than or equal to one. Using the Perron-Frobenius theorem, we know that there is exactly one eigenvalue equal to one, so that the steady state solution, *v*, solves $v=P_{m\tau }v$[36]. Recall that $v^{s_{i}}$ is defined as the subset of *v* contained in *s*_*i*_. Then, because each element of the steady state vector and stochastic matrix is positive,

$$\begin{array}{c} \left|\right|v^{u_{i}}{\left|\right|}_{\infty }=||\cdots +P_{m\tau }^{n_{i}^{+}\to u_{i}}v^{n_{i}^{+}}+P_{m\tau }^{u_{i}\to u_{i}}v^{u_{i}}+P_{m\tau }^{n_{i+1}^{-}\to u_{i}}v^{n_{i+1}^{-}}+\ldots {\left|\right|}_{\infty }. \end{array}$$ (32)

The individual terms of Eq (32), for instance, $P_{m\tau }^{n_{i}^{+}\to u_{i}}v^{n_{i}^{+}}$ represent the steady state probability density that is mapped from $n_{i}^{+}$ to *u*_*i*_ upon one iteration of the stochastic map. We note that from the conditions 3a, 3b, and 3c, only oscillators which start in *u*_*i*_ can map to the interior of *u*_*i*_. This implies that we may choose *ϵ* small enough so that the probability of transitioning from anywhere outside of *u*_*i*_ to *u*_*i*_ will be $O(\epsilon_{1}/n)$ where *n* is the maximum length over all *i* of *u*_*i*_. This implies $\left|\right|P_{m\tau }^{a\to u_{i}}{\left|\right|}_{\infty }\leq O\left(\epsilon_{1}/n\right)$ for *a* ≠ *u*_*i*_. Finally, using properties of matrix norms with Eq (32), we have

$$\begin{array}{cc} \left|\right|v^{u_{i}}{\left|\right|}_{\infty } & \leq \left|\right|P_{m\tau }^{u_{i}\to u_{i}}v^{u_{i}}{\left|\right|}_{\infty }+O\left(\epsilon_{1}/n\right).\\ & \leq \left|\right|P_{m\tau }^{u_{i}\to u_{i}}{\left|\right|}_{\infty }\cdot \left|\right|v^{u_{i}}{\left|\right|}_{\infty }+O\left(\epsilon_{1}/n\right)\\ \Rightarrow \left|\right|v^{u_{i}}{\left|\right|}_{\infty } & \leq \frac{O(\epsilon_{1}/n)}{1-\left|\right|P_{m\tau }^{u_{i}\to u_{i}}{\left|\right|}_{\infty }}. \end{array}$$ (33)

Note that the final line results from rearranging the second line using the fact that $\left|\right|P_{m\tau }^{u_{i}\to u_{i}}{\left|\right|}_{\infty }<1$. It follows immediately from properties of matrix norms that

$$\begin{array}{c} \left|\right|v^{u_{i}}{\left|\right|}_{1}\leq O\left(\epsilon_{1}\right), \end{array}$$ (34)

in other words, given *ϵ*_1_ ≪ 1, we may choose *ϵ* small enough so that once the distribution reaches its steady state, the probability that an oscillator can be found in a given region *u*_*i*_ is of order *ϵ*_1_.

#### Bounding between stable and unstable fixed points

For $\theta_{i}\in (n_{1}^{+}\cup n_{1}^{-}\cup \cdots \cup n_{k}^{+}\cup n_{k}^{-})$, let $\beta =\min|F(\theta_{i})|$, i.e., if an oscillator is expected to be found in either $n_{i}^{+}$ or $n_{i}^{-}$, it will move at least *β* closer to *s*_*i*_. Let $\kappa =\max_{i}(d\left(x,s_{i}\right)|x\in n_{i}^{+}\cup n_{i}^{-})$ where *d*(*a*, *b*) is the distance between the sets *a* and *b*. Consider oscillator *j* with initial condition $\theta_{j}\left(0\right)\in n_{i}^{+}\cup n_{i}^{-}\cup s_{i}$. Let *c* ≡ ⌈*κ*/*β*⌉. From eq (23), *E*[*θ*_*j*_(*cmτ*)] = *g* ^(*cm*)^(*θ*_*j*_(0)). The oscillator can be at most *κ* away from *s*_*i*_ and will either move at least *β* closer to *s*_*i*_, or will move inside *s*_*i*_ upon each iteration of *g* ^(*m*)^. Thus, after *c* iterations of *g* ^(*m*)^, the oscillator is expected to be in *s*_*i*_, and by condition 3b, *E*[*θ*_*j*_((*c* + 1)*mτ*)] will be in the interior of *s*_*i*_. From Eq (24), the variance is proportional to *ϵ* ^2^, and therefore, we may then choose *ϵ* small enough so that an oscillator starting in $n_{i}^{+}$ or $n_{i}^{-}$ will be mapped with probability $1-O(\epsilon_{1})$ to a location in the interior of *s*_*i*_. Furthermore, from condition 3b, any oscillator starting in *s*_*i*_ will be expected to be found in the interior *s*_*i*_, we can choose *ϵ* small enough so that any oscillator starting in *s*_*i*_ will be mapped with probability $1-O(\epsilon_{1})$ to a location in the interior of *s*_*i*_.

We have shown that by choosing *ϵ* small enough, any oscillator which starts in $n_{i}^{+}\cup n_{i}^{-}\cup s_{i}$ will map to *s*_*i*_ with probability $1-O(\epsilon_{1})$. Furthermore, from the previous section, we showed that for a small enough choice of *ϵ*, the steady state probability density contained in *u*_*i*_ will be at most $O(\epsilon_{1})$ for all *i*. Using these results, and recalling that the steady state solution of the stochastic system also solves $v=P_{(c+1)m\tau }v$, and using the same partitioning of *v* as we did in the previous section, we will consider $v^{s_{i}}$, the proportion of the steady state probability density contained in *s*_*i*_:

$$\begin{array}{cc} \left|\right|v^{s_{i}}{\left|\right|}_{1} & =\left|\right|P_{(c+1)m\tau }^{n_{i}^{+}\to s_{i}}v^{n_{i}^{+}}{\left|\right|}_{1}+\left|\right|P_{(c+1)m\tau }^{n_{i}^{-}\to s_{i}}v^{n_{i}^{-}}{\left|\right|}_{1}+\left|\right|P_{(c+1)m\tau }^{s_{i}\to s_{i}}v^{s_{i}}{\left|\right|}_{1}+O\left(\epsilon_{1}\right),\\ \left|\right|v^{s_{i}}{\left|\right|}_{1} & =\left(\left|\right|v^{n_{i}^{+}}{\left|\right|}_{1}-O\left(\epsilon_{1}\right)\right)+\left.\left(\left|\right|v^{n_{i}^{-}}{\left|\right|}_{1}-O\left(\epsilon_{1}\right)\right)+(\left|\right|v^{s_{i}}{\left|\right|}_{1}-O\left(\epsilon_{1}\right)\right)+O\left(\epsilon_{1}\right),\\ O(\epsilon_{1}) & =\left|\right|v^{n_{i}^{+}}{\left|\right|}_{1}+\left|\right|v^{n_{i}^{-}}{\left|\right|}_{1}. \end{array}$$ (35)

In other words, for the steady state distribution *v* only $O(\epsilon_{1})$ of the overall population will be found inside $n_{i}^{+}$ and $n_{i}^{-}$.

#### Neurons are found in clusters near stable fixed points with nearly identical probabilities

We have shown that if we take *ϵ* small enough, for all *i*, the probability that we will find a randomly chosen oscillator in regions *u*_*i*_, $n_{i}^{+}$ and $n_{i}^{-}$ will be at most $O(\epsilon_{1})$. Consequently, the majority of the probability distribution will be contained in the regions near stable fixed points. Here we show that the probability that an oscillator will be found in *s*_*i*_ is nearly equal to the probability that the oscillator will be found in *s*_*j*_ for any *i* and *j*.

Suppose, without loss of generality that in the absence of noise, upon successive iterations of the map *g* ^(1)^ the period *m* orbit is *θ*_*s*_1__ → *θ*_*s*_2__ → ⋯ → *θ*_*s*_*m*__ → *θ*_*s*_1__. Recalling that *τ* is the period of the external pulsing, we are interested in $P_{\tau }$, the matrix approximation to the Frobenius Perron operator, which characterizes the time evolution of the system’s probability density at intervals of *τ*. Let *γ* = mod(*i*, *m*) + 1. By condition 3d, for *θ* ∈ *s*_*i*_, *g* ^(1)^(*θ*)∈ the interior of $n_{\gamma }^{-}\cup s_{\gamma }\cup n_{\gamma }^{+}$. Recall from Eq (23) that for an oscillator with phase *θ*, its probability distribution will be centered around *g* ^(1)^(*θ*) after a time *τ* has elapsed, we may choose *ϵ* small enough so that after *τ* has elapsed, the probability of transitioning from *s*_*i*_ to $n_{\gamma }^{-}\cup s_{\gamma }\cup n_{\gamma }^{+}$ is $1-O(\epsilon_{1})$. When the distribution reaches steady state, this implies,

$$\begin{array}{cc} \left|\right|v^{s_{\gamma }}{\left|\right|}_{1} & =\cdots +\left|\right|P_{\tau }^{n_{i}^{-}\to s_{\gamma }}v^{n_{i}^{-}}{\left|\right|}_{1}+\left|\right|P_{\tau }^{s_{i}\to s_{\gamma }}v^{s_{i}}{\left|\right|}_{1}+\left|\right|P_{\tau }^{n_{i}^{+}\to s_{\gamma }}v^{n_{i}^{+}}{\left|\right|}_{1}+\cdots \\ & =O\left(\epsilon_{1}\right)+\left|\right|P_{\tau }^{s_{i}\to s_{\gamma }}v^{s_{i}}{\left|\right|}_{1}\\ & =O\left(\epsilon_{1}\right)+\left(\left|\right|v^{s_{i}}{\left|\right|}_{1}-\left|\right|P_{\tau }^{s_{i}\to n_{\gamma }^{-}}v^{s_{i}}{\left|\right|}_{1}-\left|\right|P_{\tau }^{s_{i}\to n_{\gamma }^{+}}v^{s_{i}}{\left|\right|}_{1}-O\left(\epsilon_{1}\right)\right). \end{array}$$ (36)

Note that in the second line, we have used the fact that $\left|\right|v^{n_{i}^{+}}{\left|\right|}_{1}$, $\left|\right|v^{n_{i}^{-}}{\left|\right|}_{1}$, and $\left|\right|v^{u_{i}}{\left|\right|}_{1}$ are $O(\epsilon_{1})$ terms for all *i*. In the last line, we have used the fact the sum of the probabilities of an oscillator starting in *s*_*i*_ and mapping to either $n_{\gamma }^{+}$ (represented by $\left|\right|P_{\tau }^{s_{i}\to n_{\gamma }^{+}}v^{s_{i}}{\left|\right|}_{1}$), $n_{\gamma }^{-}$ (represented by $\left|\right|P_{\tau }^{s_{i}\to n_{\gamma }^{-}}v^{s_{i}}{\left|\right|}_{1}$), or *s*_*γ*_ (represented by $\left|\right|P_{\tau }^{s_{i}\to s_{\gamma }}v^{s_{i}}{\left|\right|}_{1}$) equals $1-O(\epsilon_{1}$).

Next, from the previous section, we know that $\left|\right|v^{n_{\gamma }^{+}}{\left|\right|}_{1}=O\left(\epsilon_{1}\right)$. Recalling that the steady state solution of the stochastic system, *v*, solves $v=P_{\tau }v$ we will consider the proportion of the probability density contained in $n_{\gamma }^{+}$. Noting that because all of the entries of $P_{\tau }$ and *v* are positive, we may write

$$\begin{array}{cc} O(\epsilon_{1}) & =\left|\right|v^{n_{\gamma }^{+}}{\left|\right|}_{1}\\ & =\cdots +\left|\right|P_{\tau }^{n_{i}^{-}\to n_{\gamma }^{+}}v^{n_{i}^{-}}{\left|\right|}_{1}+\left|\right|P_{\tau }^{s_{i}\to n_{\gamma }^{+}}v^{s_{i}}{\left|\right|}_{1}+\left|\right|P_{\tau }^{n_{i}^{+}\to n_{\gamma }^{+}}v^{n_{i}^{+}}{\left|\right|}_{1}+\ldots \\ & \geq \left|\right|P_{\tau }^{s_{i}\to n_{\gamma }^{+}}v^{s_{i}}{\left|\right|}_{1}. \end{array}$$ (37)

Using an identical procedure, we can formulate the bound $O\left(\epsilon_{1}\right)\geq \left|\right|P_{\tau }^{s_{i}\to n_{\gamma }^{-}}v^{s_{i}}{\left|\right|}_{1}$. With these bounds we may rewrite Eq (36) as

$$\begin{array}{c} \left|\right|v^{s_{\gamma }}{\left|\right|}_{1}=\left|\right|v^{s_{i}}{\left|\right|}_{1}+O\left(\epsilon_{1}\right). \end{array}$$ (38)

Applying eq (38) to each region *s*_*i*_ yields

$$\begin{array}{cc} \left|\right|v^{s_{2}}{\left|\right|}_{1} & =\left|\right|v^{s_{1}}{\left|\right|}_{1}+O\left(\epsilon_{1}\right)\\ \left|\right|v^{s_{3}}{\left|\right|}_{1} & =\left|\right|v^{s_{2}}{\left|\right|}_{1}+O\left(\epsilon_{1}\right)\\ & \vdots \\ \left|\right|v^{s_{1}}{\left|\right|}_{1} & =\left|\right|v^{s_{k}}{\left|\right|}_{1}+O\left(\epsilon_{1}\right). \end{array}$$ (39)

Here, the sum of the $O(\epsilon_{1})$ terms represents the probability that a given oscillator will be found in a region without a stable fixed point of *g* ^(*m*)^. Finally, using Eq (39), and the fact that *v* is positive, one can show that for any $v^{s_{i}}$ and $v^{s_{j}}$,

$$\begin{array}{c} \left|\right|v^{s_{i}}{\left|\right|}_{1}-\left|\right|v^{s_{j}}{\left|\right|}_{1}\leq O\left(\epsilon_{1}\right), \end{array}$$ (40)

which completes the proof.

### Clustered Desynchronization with Common Periodic Perturbations

We consider a modified version of Eq (2) where each neuron feels a small, common periodic perturbation

$$\begin{array}{c} {\dot{\theta }}_{i}=\omega +\epsilon f\left(\theta_{i}\right)\delta \left(\text{mod}\left(t,\tau \right)\right)+\epsilon p\left(\omega_{1}t\right)Z\left(\theta_{i}\right)+\epsilon \eta_{i}\left(t\right)Z\left(\theta_{i}\right)+O\left(\epsilon^{2}\right),\;i=1,\ldots ,N. \end{array}$$ (41)

Here, *p*(*ω*_1_ *t*) is a periodic perturbation with period *T*_1_ = 1/*ω*_1_ common to each oscillator. This perturbation may represent the effect of coupling from an external population of neurons or coupling between neurons in the population under study. Here we give conditions for which Eq (41) exhibits clustered desynchronization.

Defining *ϕ*_*i*_ ≡ *θ*_*i*_ − *ω*_1_ *t* allows us to selectively average the term associated with *p*(*ω*_1_ *t*) from Eq (41) [54], c.f. [55]:

$$\begin{array}{c} {\dot{\vartheta }}_{i}=\omega +\epsilon f\left(\vartheta_{i}\right)\delta \left(\text{mod}\left(t,\tau \right)\right)+\epsilon G\left(\vartheta_{i}-\omega_{1}t\right)+\epsilon \eta_{i}\left(t\right)Z\left(\vartheta_{i}\right)+O\left(\epsilon^{2}\right), \end{array}$$ (42)

where $G\left(\varphi \right)=\int_{0}^{T_{1}}[p\left(\omega_{1}t\right)Z\left(\varphi_{i}+\omega_{1}t\right)]dt$ and *φ*_*i*_ = *ϑ*_*i*_ − *ω*_1_ *t*. Here *ϑ*_*i*_ is a close approximation to *θ*_*i*_ so that *φ*_*i*_ ≈ *ϕ*_*i*_. Noting that *G* is also *T*_1_ periodic, we selectively average Eq (42) to yield

$$\begin{array}{c} {\dot{\Theta }}_{i}=\omega +\epsilon K+\epsilon f\left(\Theta_{i}\right)\delta \left(\text{mod}\left(t,\tau \right)\right)+\epsilon \eta_{i}\left(t\right)Z\left(\Theta_{i}\right)+O\left(\epsilon^{2}\right), \end{array}$$ (43)

Here, Θ_*i*_ ≈ *ϑ*_*i*_ and $K=\int_{0}^{T_{1}}G\left(\varphi_{i}-\omega_{1}t\right)dt$. Notice that Eq (43) is in an identical form as Eq (2) for which our main theoretical result still holds.
